# Abemaciclib and Vacuolin-1 decrease aggregate-prone TDP-43 accumulation by accelerating autophagic flux

**DOI:** 10.1016/j.bbrep.2024.101705

**Published:** 2024-04-01

**Authors:** Yoshinori Tanaka, Lina Kozuma, Hirotsugu Hino, Kosuke Takeya, Masumi Eto

**Affiliations:** a Biochemistry Unit, Faculty of Veterinary Medicine, Okayama University of Science, Imabari-shi, Ehime, Japan; b Division of Anatomical Science, Department of Functional Morphology, Nihon University School of Medicine, Itabashi-ku, Tokyo, Japan

**Keywords:** Abemaciclib, Vacuolin-1, Autophagic flux, TDP-43, PI(3)P

## Abstract

(Macro)autophagy is a cellular degradation system for unnecessary materials, such as aggregate-prone TDP-43, a central molecule in neurodegenerative diseases including amyotrophic lateral sclerosis and frontotemporal lobar degeneration. Abemaciclib (Abe) and vacuolin-1 (Vac) treatments are known to induce vacuoles characterized by an autophagosome and a lysosome component, suggesting that they facilitate autophagosome-lysosome fusion. However, it remains unknown whether Abe and Vac suppress the accumulation of aggregate-prone TDP-43 by accelerating autophagic flux. In the present study, the Abe and Vac treatment dose-dependently reduced the GFP/RFP ratio in SH-SY5Y neuroblastoma cells stably expressing the autophagic flux marker GFP-LC3-RFP-LC3ΔG. Abe and Vac also increased the omegasome marker GFP-ATG13 signal and the autophagosome marker mCherry-LC3 localized to the lysosome marker LAMP1-GFP. The Abe and Vac treatment decreased the intracellular level of the lysosome marker LAMP1-GFP in SH-SY5Y cells stably expressing LAMP1-GFP, but did not increase the levels of LAMP1-GFP, the autophagosome marker LC3-II, or the multivesicular body marker TSG101 in the extracellular vesicle-enriched fraction. Moreover, Abe and Vac treatment autophagy-dependently inhibited GFP-tagged aggregate-prone TDP-43 accumulation. The results of a PI(3)P reporter assay using the fluorescent protein tagged-2 × FYVE and LAMP1-GFP indicated that Abe and Vac increased the intensity of the PI(3)P signal on lysosomes. A treatment with the VPS34 inhibitor wortmannin (WM) suppressed Abe-/Vac-facilitated autophagic flux and the degradation of GFP-tagged aggregate-prone TDP-43. Collectively, these results suggest that Abe and Vac degrade aggregate-prone TDP-43 by accelerating autophagosome formation and autophagosome-lysosome fusion through the formation of PI(3)P.

## Highlights

• Abe and Vac accelerate autophagic flux.
• Abe and Vac do not increase release of extracellular vesicles.
• Abe and Vac autophagy-dependently suppress aggregate-prone TDP-43 accumulation.
• Acceleration of autophagic flux by Abe and Vac is mediated by PI(3)P formation.

## 1 Introduction

Macroautophagy (hereafter autophagy) is a conserved cellular degradation system involving the cytosolic double-membraned vesicle, the autophagosome. Autophagosome formation starts from an omegasome that serves as the site of nucleation for a membrane cistern called a phagophore, which grows around a specific portion of the cytoplasm and isolates unnecessary materials within the autophagosome. The organelle then fuses with a lysosome to form an autolysosome for the degradation of its contents. A reduction in autophagic activity has been implicated in the development of multiple diseases, including neurodegenerative diseases [1].

The nuclear protein TDP-43 is a central molecule in the pathogenesis of most cases of amyotrophic lateral sclerosis (ALS) and approximately 50% of those of frontotemporal lobar degeneration (FTLD-TDP). The aggregation of TDP-43 in the cytoplasm induces neurodegeneration through a loss-of-function mechanism in the nucleus and gain-of-toxic-function mechanism in the cytoplasm [2]. Aggregated TDP-43 in the brains of patients has a prion-like property, which induces the seed-dependent accumulation of TDP-43 throughout the brain [3,4]. Autophagy was identified as the main degradation system for aggregate-prone TDP-43, and the suppression of autophagic flux increased aggregate-prone TDP-43 accumulation [5,6].

We previously showed that the CDK4/6 inhibitor abemaciclib (Abe) and the PIKfyve inhibitor vacuolin-1 (Vac) induced vacuole-like autolysosome formation [7]. The effects of these drugs were facilitated by inducers of autophagosome-lysosome fusion and suppressed by inhibitors of autophagosome-lysosome fusion, suggesting that the vacuoles formed by Abe and Vac were due to the facilitation of autophagosome-lysosome fusion. On the other hand, an autophagic flux assay using the autophagic flux reporter GFP-LC3-RFP-LC3ΔG [8] on the lung cancer cell-line A549 indicated that a high concentration of Abe delayed autophagic flux [9]. Therefore, it currently remains unknown whether Abe and Vac facilitate autophagic flux or if they exert opposing effects at low or high concentrations on autophagic flux.

In the present study, we investigated the effects of Abe and Vac on autophagic flux in the human neuroblastoma cell-line SH-SY5Y. Abe and Vac increased omegasome formation and autolysosome formation, and decreased lysosome marker levels without increasing their extracellular levels. Abe and Vac also autophagy-dependently suppressed GFP-tagged aggregate-prone TDP-43 accumulation. Moreover, increased PI(3)P levels were associated with the acceleration of autophagic flux and degradation of aggregate-prone TDP-43 by Abe and Vac. Collectively, the results obtained herein indicate that Abe and Vac decreased aggregate-prone TDP-43 accumulation by facilitating autophagosome-lysosome fusion through the formation of PI(3)P.

## 2 Materials and methods

### 2.1 Antibodies and reagents

The antibodies and reagents used in the present study are listed in Supplementary Table.

### 2.2 Expression vectors

pCMV-GFP-LC3-RFP-LC3ΔG was used as previously reported [6]. pGFP-tagged C-terminal fragment of TDP-43 (TDP-43 amino acids (aa) 162–414) was gifted by Dr. Takashi Nonaka (TMIMS, Tokyo, Japan) [10]. pLentiN-LAMP1-GFP and pLentiN-mCherry-LC3 was gifted by Dr. Naoharu Takano [11]. pMD2.G (#12259 from Didier Trono Lab), psPAX2 (#12260 from Didier Trono Lab), pEGFP-C1-hATG13 (#22875 from Noboru Mizushima Lab), LAMP1-mGFP (#34831 from Dell’Angelica Lab), pEGFP-2 × FYVE (#140047 from Harald Stenmark Lab), and pmCherry-2 × FYVE (#140050 from Harald Stenmark Lab) were purchased from Addgene.

### 2.3 Lentiviral production

Lentivirus production was performed as previously reported [11]. Briefly, lentiviruses were produced in Lenti-X 293T cells (Takara Bio, Shiga, Japan) by transfection of pMD2.G and psPAX2 with pLentiN-LAMP1-GFP or pLentiN-mCherry-LC3 using TransIT-Lenti Transfection Reagent (#MIR6600; Mirus, WI, USA). After 48 h, viral supernatants were filtered by a PVDF 0.45 μm filter (Millex-HV, Millipore, MA, USA).

### 2.4 Generation of stable cell lines

To generate SH-SY5Y cell stably expressing GFP-LC3-RFP-LC3ΔG, SH-SY5Y cell stably expressing LAMP1-GFP, HeLa cell stably expressing GFP-2 × FYVE, and HeLa cell stably expressing mCherry-2 × FYVE, the protocol previously described was used in this study [6]. Positive clones effectively expressing the fluorescent reporter were isolated by cell sorting with fluorescence-activated cell sorting or limiting dilution cloning in 96-well plates. SH-SY5Y cell stably expressing LAMP1-GFP and mCherry-LC3, and HeLa cell stably expressing mCherry-2 × FYVE and LAMP1-GFP were generated by the protocol described previously [11]. Briefly, the ectopic mCherry-LC3 or LAMP1-GFP was introduced into SH-SY5Y cells stably expressing LAMP1-GFP or HeLa cells stably expressing mCherry-2 × FYVE by polybrene (Nacalai Tesque, Kyoto, Japan), and subjected to selection with blasticidin (#029–18701; Fujifilm Wako) or puromycin (#19752-64; Nacalai).

### 2.5 Cell culture

SH-SY5Y and HeLa cells were provided by ATCC (Virginia, USA). SH-SY5Y cells stably expressing mRFP-LC3-GFP-LC3ΔG, ATG16L1 knockout HeLa cells, and control HeLa cells [12] were kindly gifted by David Rubinsztein's Lab (Cambridge, UK). SH-SY5Y and HeLa cells were cultured as previously reported [13].

### 2.6 Assessment of phagophore formation

SH-SY5Y cells transfected with GFP-ATG13 using TransIT-2020 reagent (#MIR5400; Mirus) were seeded at a density of 1.0 × 10^4^ cells on μ-Slide 8 Well high (ibidi, Gräfelfing, Germany). After a 24-hr incubation with 2 μM Abe, 100 nM Vac in the growth medium, or HBSS, live-cell imaging was conducted using a 60 × objective lens equipped on a LSM780 confocal laser microscope (Carl Zeiss, Germany). Signal intensity per cell was quantified using ImageJ and the Analyse Particles plugin (a constant threshold for all images per experiment was applied) of Fiji software [14]. At least 41 cells in each of the three independent experiments were subjected to counting.

### 2.7 Autolysosome analysis

An autolysosome analysis using SH-SY5Y cell stably expressing mCherry-LC3 and LAMP1-GFP was performed as previously reported [13]. Briefly, SH-SY5Y cells stably expressing mCherry-LC3 and LAMP1-GFP were seeded at a density of 1.0 × 10^4^ cells on μ-Slide 8 Well high (ibidi). After a 24-hr stimulation with 2 μM Abe or 100 nM Vac, live-cell imaging was conducted using a 60 × objective lens equipped on a LSM780 confocal laser microscope (Carl Zeiss). After setting thresholds for the red (mCherry-LC3) and green (LAMP1-GFP) signals, the signal intensity of mCherry-LC3 and the number of mCherry-LC3 positive dot were calculated by the Analyse Particles plugin on Fiji software [14]. To measure the signal intensity of the double-positive area for mCherry-LC3 and LAMP1-GFP, red and green binary images were overlapped using the paste control “AND”, and signal intensity was calculated by the Analyse Particles plugin. The ratio of mCherry-LC3 co-localized to LAMP1-GFP was calculated from the signal intensity double-positive for red and green divided by the total red signal intensity. At least 42 cells in each of the three independent experiments were subjected to counting.

An autolysosome analysis with DALGreen (DALG) staining was performed as previously reported [13]. Briefly, HeLa cells were seeded at a density of 1.0 × 10^4^ cells on μ-Slide 8 Well high (ibidi). After a 24-hr stimulation with 2 μM Abe, 100 nM Vac, or 100 nM Baf, live-cell imaging was conducted using a 60 × objective lens equipped on a LSM780 confocal laser microscope (Carl Zeiss). Signal intensity per cell was quantified using ImageJ and the Analyse Particles plugin (a constant threshold for all images per experiment was applied) of Fiji software [14]. At least 61 cells in each of the three independent experiments were subjected to counting.

### 2.8 Immunoblot analysis

An immunoblot analysis was conducted as described in a previous study [13]. Cells were lysed for 20 min with 1% (w/v) Sarkosyl in A68 [10 mM Tris-HCl buffer, pH 7.5, 0.8 M NaCl, 1 mM ethylene glycol bis(β-aminoethyl ether)*-N,N,N,N*-tetraacetic acid (EGTA)] on ice. The lysates were collected and the protein concentration was measured using BCA Protein Assay Kit (Thermo Fisher Scientific), and SDS-sample buffer was added to give a final concentration of 2% SDS. Samples were boiled for 10 min and subjected to SDS-PAGE, and proteins were transferred onto polyvinylidene difluoride membrane (Millipore). The blots were blocked with Blocking One (Nacalai), and incubated overnight with the indicated primary antibody in Tris-buffer containing 10% (v/v) calf serum at RT. The membranes were washed, and incubated with a biotin-conjugated secondary antibody (Vector) for 2 h at RT. Signals were detected using the ABC staining kit (Vector). The digitized images were analyzed using Fiji [14].

### 2.9 Preparation of cellular and extracellular vesicle fractions

Cellular and extracellular vesicle (EV) fractions for the immunoblot analysis were prepared as shown in a previous study [13]. To prepare the cellular fraction, cells were washed with cold PBS, detached from the dish by trypsinization, collected into a 15-mL tube, and centrifuged at 3000×*g* at 4 °C for 10 min. The cells in the pellet were lysed with 1% (w/v) Sarkosyl in A68 by sonication. Protein concentrations in cell lysates were estimated using a BCA Protein Assay Kit (Thermo Fisher Scientific), and SDS-sample buffer was added to give a final concentration of 1% SDS. Regarding the fractionation of EV in culture medium, culture medium collected from the 10-cm dish was pre-cleared by centrifugation at 1000×*g* at 4 °C for 10 min to remove nuclei and cell debris. The pre-cleared medium was pooled in a centrifugation tube (361706, Beckman Coulter, CA, USA) and centrifuged at 20,000×*g* at 4 °C for 1 h. The pellet was collected as the P1 fraction and lysed with 50 μL of 2% SDS-sample buffer. Supernatants were recollected in the centrifugation tube (344059, Beckman Coulter) and centrifuged for 1 h at 110,000×*g* at 4 °C. The pellet was collected as the P2 fraction and lysed with 50 μL of 2% SDS-sample buffer. Samples were boiled for 10 min and subjected to immunoblot analysis.

### 2.10 Quantification of TDP-43 aggregate formation

TDP-43 aggregate formation was quantified as previously described [6]. TDP-43 aa: 162–414 aggregates in SH-SY5Y cells cultured in 12-well plate was monitored under a 20 × objective lens equipped with the BZ-X710 fluorescence microscope. TDP-43 aa: 162–414 aggregates were identified with the GFP signal in short exposure images. At least five different frames of images were captured and at least 289 cells were counted per experiment. The proportion of cells with at least one aggregate was scored as a ratio of GFP-positive cells. Three independent experiments were performed for quantification.

### 2.11 Quantification of GFP-2 × FYVE signal intensity

HeLa cells stably expressing GFP-2 × FYVE were seeded at a density of 1.0 × 10^4^ cells on μ-Slide 8 Well high (ibidi). After a 24-hr stimulation with 2 μM Abe or 100 nM Vac with or without 1 μM WM, live-cell imaging was conducted using a 60 × objective lens equipped on a LSM780 confocal laser microscope (Carl Zeiss). Signal intensity per cell was quantified using ImageJ and the Analyse Particles plugin (a constant threshold for all images per experiment was applied) of Fiji software [14]. At least 20 cells in each of the three independent experiments were subjected to counting.

### 2.12 Localization of mCherry-2 × FYVE signal on LAMP1-GFP signal

HeLa cells stably expressing mCherry-2 × FYVE and LAMP1-GFP were seeded at a density of 1.0 × 10^4^ cells on μ-Slide 8 Well high (ibidi). After a 24-hr stimulation with 2 μM Abe or 100 nM Vac, live-cell imaging was conducted using a 60 × objective lens equipped on a LSM780 confocal laser microscope (Carl Zeiss). After setting thresholds for the red (mCherry-2 × FYVE) and green (LAMP1-GFP) signals, the signal intensity of mCherry-2 × FYVE was calculated by the Analyse Particles plugin of Fiji software [14]. To measure the signal intensity of the double-positive area for mCherry-2 × FYVE and LAMP1-GFP, red and green binary images were overlapped using the paste control “AND”, and signal intensity was calculated by the Analyse Particles plugin. The ratio of mCherry-2 × FYVE co-localized to LAMP1-GFP was calculated from the signal intensity double-positive for red and green divided by the total red signal intensity. At least 23 cells in each of the three independent experiments were subjected to counting.

### 2.13 siRNA transfection

Cells were counted using the TC20 automated cell counter (Bio-rad) and seeded at a density of 0.5 × 10^5^ cells on 12-well plates. In knockdown experiments, cells were transfected with 20 pmol siRNA using Lipofectamine RNAiMAX (#13778; Invitrogen). Predesigned siRNA duplexes (Thermo Fisher Scientific) to target human PIKfyve#1 (HSS140704: forward, 5′-GGA AAG GAA UUA GUC AAC UGG CUA A-3’; reverse, 5′-UUA GCC AGU UGA CUA AUU CCU UUC C-3′) and PIKfyve#2 (HSS140705: forward, 5′-GGA GAC CUC CGA GCU UGC ACA UAU U -3’; reverse, 5′-AAU AUG UGC AAG CUC GGA GGU CUC C-3′) were used for human PIKfyve knockdown. Stealth RNAi Negative Control Duplexes, Med GC (#12935300, Thermo Fisher Scientific) was used as a negative control of siRNA.

### 2.14 Statistical analysis

Data were analyzed with an unpaired two-tailed Student's *t*-test or the Mann-Whitney *U* test powered by Excel (Microsoft Office) or EZR [15]. Results are expressed as means ± SEM. P values < 0.05 were considered to be significant.

## 3 Results

### 3.1 Abe and Vac facilitate autophagic flux in SH-SY5Y cells

**Fig. 1:**
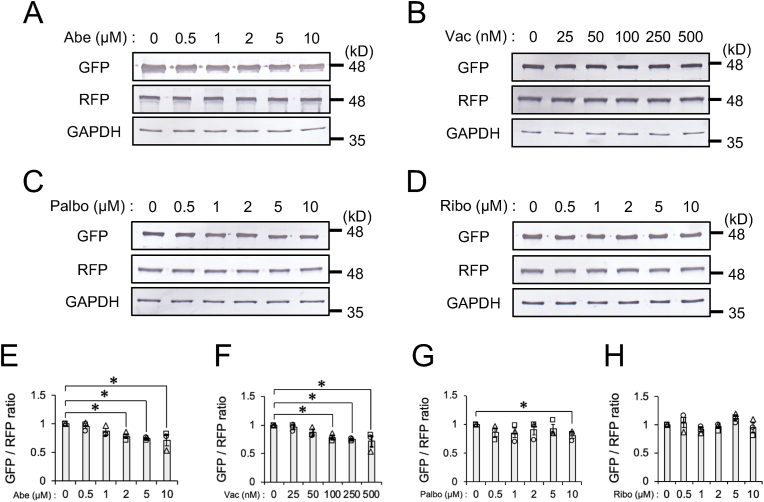
Dose-dependent effects of Abe and Vac on autophagic flux SH-SY5Y cells stably expressing GFP-LC3-RFP-LC3ΔG were exposed to the indicated concentration of (**A**) Abe, (**B**) Vac, (**C**) Palbo, or (**D**) Ribo for 24 h. Representative immunoblots (GFP, RFP, and GAPDH) are shown. (**E-H**) Densitometric data on GFP and RFP were obtained from immunoblotting results, and the GFP/RFP ratio in cells exposed to (**E**) Abe, (**F**) Vac, (**G**) Palbo, or (**H**) Ribo was calculated from densitometric data. The GFP/RFP ratio normalized against cells exposed to DMSO is shown. Bar graph data are presented as means ± S.E.M. (*N* = 3). * indicates P < 0.05 by the two-tailed unpaired *t*-test.

To establish whether Abe and Vac facilitate autophagic flux in neuronal cells, we prepared SH-SY5Y neuroblastoma cells stably expressing the autophagic flux reporter, GFP-LC3-RFP-LC3ΔG. GFP-LC3-RFP-LC3ΔG expressed in cells is cleaved into the autophagy marker GFP-LC3 and the internal control RFP-LC3ΔG at a ratio of 1:1 [8]. Therefore, a decrease in the GFP/RFP ratio indicates the acceleration of autophagic flux. SH-SY5Y cells stably expressing GFP-LC3-RFP-LC3ΔG were exposed to Abe, Palbociclib (Palbo), or Ribociclib (Ribo), inhibitors of CDK4/6, or Vac, an inhibitor of PIKfyve, for 24 h. Abe and Vac significantly decreased the GFP/RFP ratio in a dose-dependent manner (Abe: Fig. 1A and E, Vac: Fig. 1B and F) as observed in HBSS treatment (Supplementary Figs. 1A–B), suggesting that they accelerated autophagic flux in SH-SY5Y cells. On the other hand, only 10 μM of Palbo significantly decreased the GFP/RFP ratio (Fig. 1C and G), while Ribo at the tested concentrations did not significantly affect the GFP/RFP ratio (Fig. 1D and H). The effects of these agents on the autophagic flux were consistent to their potencies for inducing vacuole formation [9]. These results suggest that Abe and Vac facilitate autophagic flux, and the effects of Abe overlapped those of Vac in the regulation of autophagic flux.

**Fig. 2:**
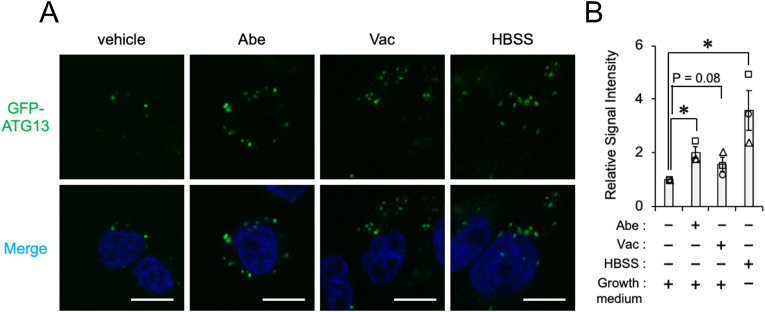
The influence of Abe and Vac on phagophore formation SH-SY5Y cells transfected with the GFP-tagged ATG13 (GFP-ATG13) were exposed to vehicle (DMSO), 2 μM Abe, 100 nM Vac, or HBSS for 24 h. (**A**) Representative confocal live-cell images are shown. Nuclei (Blue) were stained with Hoechst33342. Scale bar: 10 μm. (**B**) The relative signal intensities of GFP-ATG13 normalized against cells exposed to DMSO from three independent experiments including at least 41 cells are shown. Bar graph data are presented as means ± S.E.M. (*N* = 3). * indicates P < 0.05 by the two-tailed unpaired *t*-test.

PIKfyve synthesizes phosphatidylinositol-3,5-bisphosphate [PI(3,5)P_2_] from phosphatidylinositol 3-phosphate [PI(3)P] [16]. Suppression of PIKfyve activity elevates the level of PI(3)P [17], an inducer of autophagy [16]. To investigate whether Abe and Vac regulated phagophore formation in SH-SY5Y cells, we focused on the omegasome marker ATG13. ATG13 transiently exists in the omegasome intermediate structure and is not influenced by the rate of degradation of autophagosomes [18]. Abe significantly increased GFP-ATG13 signal in SH-SY5Y cells, mimicking HBSS treatment as a positive control of autophagy induction. Vac tended to increase GFP-ATG13 signal, suggesting that Abe and, to the lesser extent, Vac increase phagophore formation in SH-SY5Y cells (Fig. 2A and B).

**Fig. 3:**
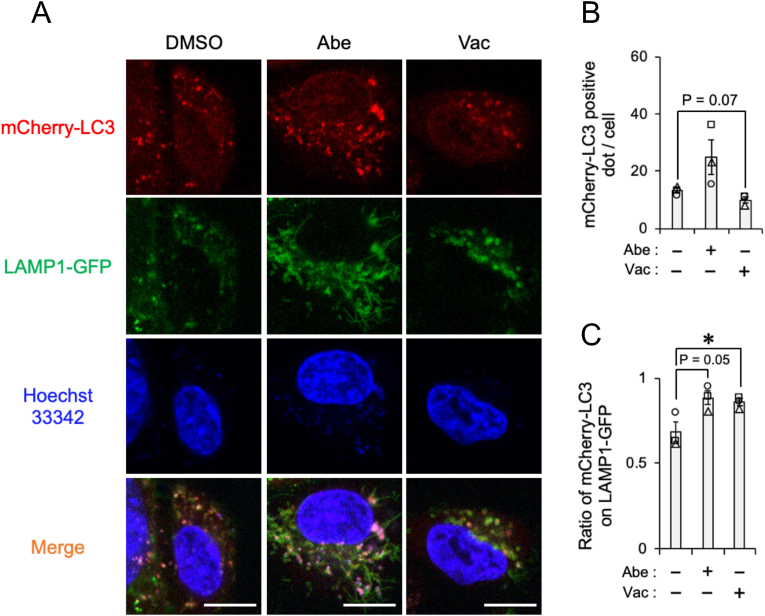
The influence of Abe and Vac on autolysosome formation SH-SY5Y cells stably expressing mCherry-LC3 and LAMP1-GFP were exposed to vehicle (DMSO), 2 μM Abe, or 100 nM Vac for 24 h. (**A**) Representative confocal live-cell images are shown. Nuclei (Blue) were stained with Hoechst33342. Scale bar: 10 μm. (**B–C**) The number of mCherry-LC3 positive dot (**B**) and the ratio of mCherry-LC3 signal on LAMP1-GFP signal to total mCherry-LC3 signal (**C**) calculated from at least 42 cells are shown. Bar graph data are presented as means ± S.E.M. (*N* = 3). * indicates P < 0.05 by the two-tailed unpaired *t*-test.

We next investigated whether Abe and Vac influence autolysosome formation using SH-SY5Y cell stably expressing the autophagosome marker mCherry-LC3 and the lysosome marker LAMP1-GFP (Fig. 3A). The number of mCherry-LC3 positive dots increased by Abe and decreased by Vac (Fig. 3B). The ratio of mCherry-LC3 localized to LAMP1-GFP that represent the autolysosome formation elevated upon either Abe or Vac treatment (Fig. 3C). Similarly, Abe and Vac increased the autolysosome marker DALG signal intensity in HeLa cells (Supplementary Figs. 2A–B). These results suggest that Abe and Vac facilitate autolysosome formation in SH-SY5Y and HeLa cells.

### 3.2 Abe and Vac do not increase the release of EV from SH-SY5Y cells

**Fig. 4:**
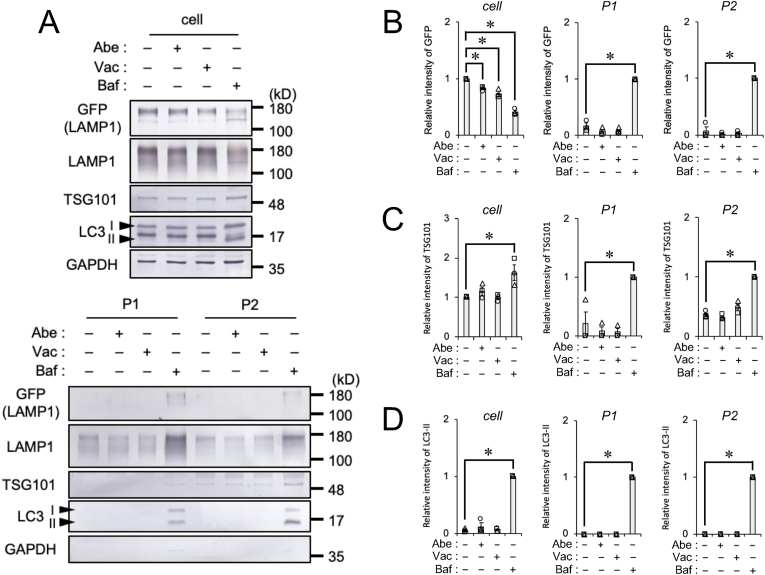
Effects of Abe and Vac on the secretion of extracellular vesicles SH-SY5Y cells stably expressing LAMP1-GFP were exposed to vehicle (DMSO), 2 μM Abe, 100 nM Vac, or 100 nM Baf for 24 h. The P1 (20,000×*g* pellet) and P2 (110,000×*g* pellet) cell fractions were prepared. (**A**) Representative immunoblots (GFP, LAMP1, TSG101, LC3, and GAPDH) of each fraction are shown. (**B-D**) Densitometric data on (**B**) GFP, (**C**) TSG101, and (**D**) LC3-II are calculated from immunoblotting results. Relative signal intensities normalized against cells exposed to DMSO or Baf are shown. Bar graph data are presented as means ± S.E.M. (*N* = 3). * indicates P < 0.05 by the two-tailed unpaired *t*-test or Mann-Whitney *U* test.

Amphisomes are degradative organelles comprised of an autophagosome and multivesicular bodies (MVB). A previous study demonstrated that the inhibition of PIKfyve induced the release of amphisomes containing TDP-43 into the extracellular space via an autophagy-regulating mechanism [19]. On the other hand, the inhibition of PIKfyve had a negative impact on the recycling of β1-integrin from endosomes or lysosomes back into the plasma membrane [20]. To investigate whether Abe and Vac increased the amount of extracellular vesicle (EV), SH-SY5Y cells stably expressing the lysosome marker LAMP1-GFP were exposed to vehicle, 2 μM Abe, 100 nM Vac, or 100 nM bafilomycin A1 (Baf), an inhibitor of autophagosome-lysosome fusion, for 24 h. To quantify the amount of EV, conditional media were subjected to sequential centrifugation at 20,000×*g* (the P1 fraction) for the collection of larger vesicles, followed by 110,000×*g* (the P2 fraction) for the collection of smaller vesicles as previously reported [13]. The expression levels of cellular proteins were quantified using the lysates of adhered cells. Immunoblotting and densitometric data showed that the cellular level of LAMP1-GFP was significantly decreased by the Abe and Vac treatment and also by the Baf treatment. The levels of the MVB marker TSG101 and the autophagosome marker LC3-II in the cell fraction were increased by the Baf treatment, but not by the Abe and Vac treatment (Fig. 4A–D). The extracellular level of LAMP1-GFP was significantly increased by the Baf treatment, but not by the Abe and Vac treatment (Fig. 4A and B). TSG101 and LC3-II levels in the extracellular fraction were increased by the Baf treatment, which is consistent with previous findings [13], but not by the Abe and Vac treatment (Fig. 4A, C-D). These results indicate that the Abe and Vac treatment decreased cellular lysosomes, but not increase the release of EV.

### 3.3 Abe and Vac suppress the accumulation of the aggregate-prone C-terminal fragment of TDP-43

**Fig. 5:**
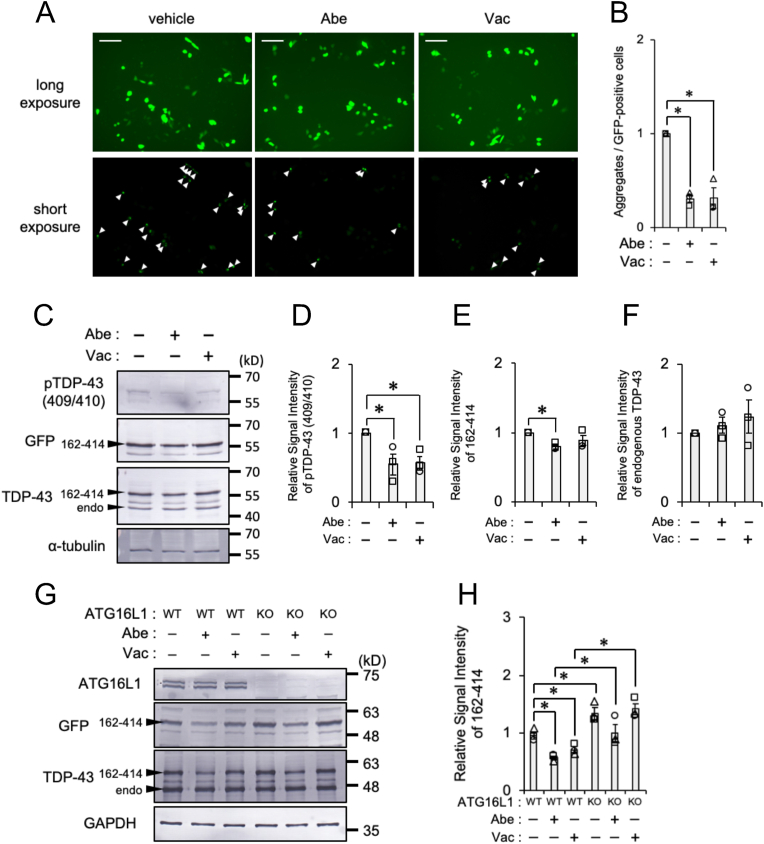
Abe-/Vac-induced autophagy-dependent degradation of aggregate-prone TDP-43 (**A-B**) SH-SY5Y cells transfected with the GFP-tagged C-terminal fragment of TDP-43 (TDP-43 aa 162–414) were exposed to vehicle (DMSO), 2 μM Abe, or 100 nM Vac for 24 h. (**A**) Representative live-cell images are shown. Long and short exposure images were captured in the same image field. Arrowheads show cells harboring TDP-43 aa 162–414 aggregates. Scale bar: 100 μm. (**B**) The ratio of cells harboring aggregates to GFP-positive cells was calculated from images including at least 289 cells. Bar graph data are presented as means ± S.E.M. (*N* = 3). * indicates P < 0.05 by the two-tailed unpaired *t*-test. (**C–F**) SH-SY5Y cells transfected with TDP-43 aa 162–414 were exposed to vehicle (DMSO), 2 μM Abe, or 200 nM Vac for 24 h. (**C**) Representative immunoblots (pTDP-43, GFP, TDP-43, and α-tubulin) are shown. Arrowheads of “162–414” indicate TDP-43 aa 162–414 bands, and arrowheads of “endo” indicates endogenous TDP-43 bands. Densitometric data on pTDP-43, TDP-43 aa 162–414, and endogenous TDP-43 are calculated from immunoblotting results. Bar graph data are presented as means ± S.E.M. (*N* = 3). * indicates P < 0.05 by the two-tailed unpaired *t*-test. (**G-H**) ATG16L1 WT and KO HeLa cells transfected with TDP-43 aa 162–414 were exposed to vehicle (DMSO), 2 μM Abe, or 100 nM Vac for 24 h. **(G)** Representative immunoblots (ATG16L1, GFP, TDP-43, and GAPDH) are shown. Arrowheads of “162–414” indicate TDP-43 aa 162–414 bands, and arrowheads of “endo” indicates endogenous TDP-43 bands. **(H)** Densitometric data on TDP-43 aa 162–414 are calculated from immunoblotting results. Relative signal intensities normalized against the average intensity in each experiment are shown. Bar graph data are presented as means ± S.E.M. (*N* = 3). * indicates P < 0.05 by the two-tailed unpaired *t*-test.

To investigate whether Abe and Vac suppress aggregate-prone TDP-43 accumulation, we examined their effects on the accumulation of aggregate-prone GFP-tagged C-terminal of TDP-43 aa: 162–414 [10]. We initially investigated whether Abe and Vac affected the aggregation of TDP-43 aa: 162–414 in SH-SY5Y cells. The solid aggregates of TDP-43 aa: 162–414 were found in some of SH-SY5Y cells (Supplementary Fig. 3). Abe and Vac significantly decreased its aggregation (Fig. 5A and B). To examine whether Abe and Vac decrease pathologically aggregated form of TDP-43, we focused on phosphorylated TDP-43 at serine 409/410 (pTDP-43), an indicator of the pathophysiologic aggregation [21]. Immunoblotting results showed that Abe and Vac significantly decreased pTDP-43 levels (Fig. 5C and D). Abe significantly decreased TDP-43 aa: 162–414 levels, but Vac did not (Fig. 5E). Then, Abe and Vac did not decrease the endogenous TDP-43 levels (Fig. 5F). These results suggest that Abe and Vac decrease pathologically aggregated form of TDP-43 via autophagy. We then prepared ATG16L1-WT and -KO (autophagy-null) HeLa cells to establish whether Abe and Vac suppressed the accumulation of aggregate-prone TDP-43 in a manner that was dependent on autophagy. The 24-hr treatment with Abe and Vac significantly decreased TDP-43 aa: 162–414 levels in ATG16L1-WT HeLa cells. On the other hand, in ATG16L1-KO HeLa cells, only Abe treatment produced a subtle (but not significant) decrease in the lower levels of TDP-43 aa: 162–414, suggesting an involvement of autophagy. TDP-43 aa: 162–414 levels in ATG16L1-KO cells were significantly higher than ATG16L1-WT cells in each treatment (Fig. 5G and H). These results suggest that Abe and Vac suppressed aggregate-prone TDP-43 accumulation via an autophagy-dependent mechanism.

### 3.4 PI(3)P formation is associated with Abe-/Vac-facilitated autophagic flux and the degradation of aggregate-prone TDP-43

**Fig. 6:**
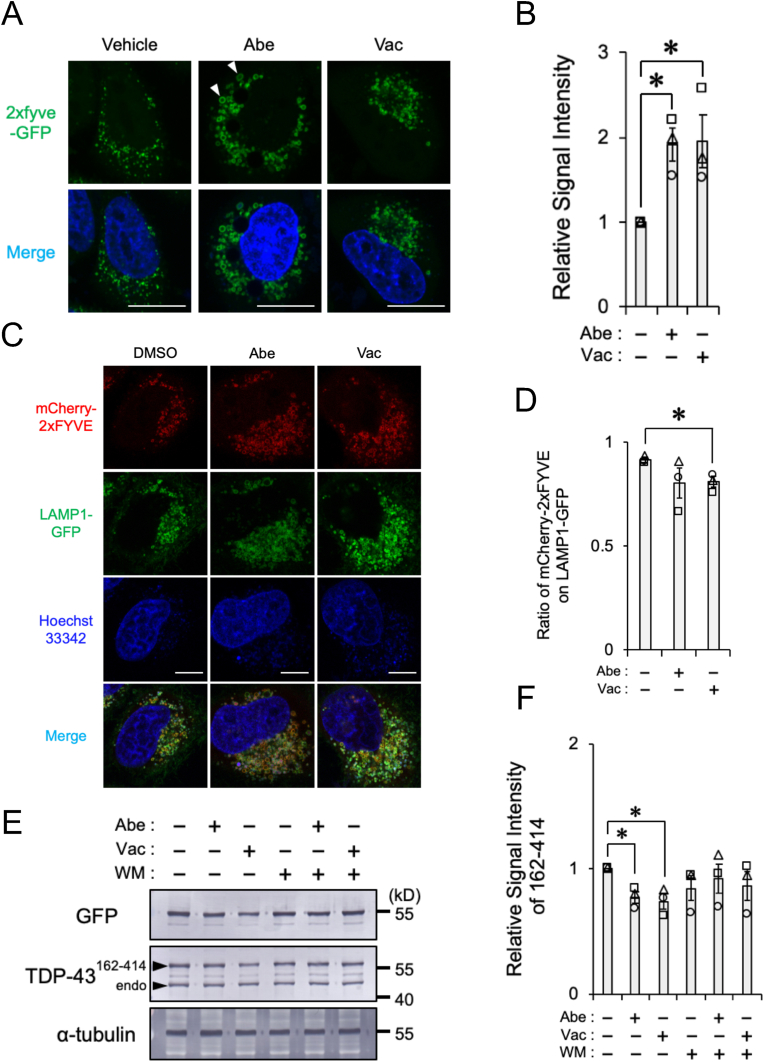
Abe-/Vac-induced PI(3)P formation associated with the facilitation of autophagic flux (**A**-**B**) HeLa cells stably expressing GFP-2 × FYVE were exposed to vehicle (DMSO), 2 μM Abe, or 100 nM Vac for 24 h. (**A**) Representative confocal live-cell images are shown. Nuclei (Blue) were stained with Hoechst33342. Scale bar: 20 μm. The arrowhead shows the localization of the GFP signal to enlarged vesicles. (**B**) The relative signal intensities of GFP-2 × FYVE normalized against cells exposed to DMSO from three independent experiments including at least 21 cells are shown. Bar graph data are presented as means ± S.E.M. (*N* = 3). * indicates P < 0.05 by the two-tailed unpaired *t*-test. (**C-D**) HeLa cells stably expressing mCherry-2xFYVE and LAMP1-GFP were exposed to vehicle (DMSO), 2 μM Abe, or 100 nM Vac for 24 h. (**C**) Representative confocal live-cell images are shown. Nuclei (Blue) were stained with Hoechst33342. Scale bar: 10 μm. (**D**) The ratio of mCherry-2xFYVE signal on LAMP1-GFP signal to total mCherry-2xFYVE signal calculated from at least 23 cells are shown. Bar graph data are presented as means ± S.E.M. (*N* = 3). * indicates P < 0.05 by the two-tailed unpaired *t*-test. (**E**–**F**) HeLa cells transfected with TDP-43 aa 162–414 were exposed to vehicle (DMSO), 2 μM Abe, 200 nM Vac, 1 μM WM, 1 μM WM plus 2 μM Abe, or 1 μM WM plus 200 nM Vac for 6 h. (**E**) Representative immunoblots (GFP, TDP-43, and α-tubulin) are shown. (**F**) Densitometric data on GFP (TDP-43 aa 162–414) are calculated from immunoblotting results. Relative signal intensities normalized against cells exposed to DMSO from three independent experiments are shown. Bar graph data are presented as means ± S.E.M. (*N* = 3). * indicates P < 0.05 by the two-tailed unpaired *t*-test.

Previous studies suggested that the inhibition of PIKfyve facilitated vesicle fusion through increases in PI(3)P levels [22]. To clarify whether Abe and Vac increased PI(3)P levels, we prepared HeLa cells stably expressing the GFP-tagged 2 × FYVE domain, a marker for PI(3)P [23]. We initially investigated whether the knockdown of *PIKFIVE* affected PI(3)P levels. Live-cell imaging showed that the knockdown of *PIKFIVE* significantly increased GFP signal intensity (Supplementary Figs. 4A–B), confirming that PIKfyve negatively regulated PI(3)P levels. We then examined the effects of Abe and Vac on PI(3)P levels. Live-cell imaging showed that the Abe and Vac treatment for 24 h significantly increased GFP signal intensity, which localized to vacuole-like vesicles (Fig. 6A and B). In HeLa cells expressing mCherry-tagged 2 × FYVE domain and LAMP1-GFP, majority of the mCherry signal was co-localized to LAMP1-GFP, indicating lysosomal localization. The PI(3)P on lysosomes significantly decreased upon Vac treatment (Fig. 6C and D). Because PI(3)P on lysosomes is required for autophagosome-lysosome fusion [24], Abe/Vac treatment causes the increases in PI(3)P levels on lysosomes, leading the formation of the vacuole-like autolysosome.

Although PI(3)P is generated from phosphatidylinositol (PI), phosphatidylinositol-3,5-bisphosphate [PI(3,5)P_2_], and phosphatidylinositol-3,4-bisphosphate [PI(3,4)P_2_] [16], the enzyme most responsible for PI(3)P synthesis is VPS34 [25]. To elucidate the mechanisms by which the inhibition of VPS34 affected PI(3)P levels in cells exposed to Abe and Vac, GFP-tagged 2 × FYVE signal intensity was examined in cells treated with vehicle, WM, an inhibitor of VPS34, Abe plus WM, or Vac plus WM for 24 h. Live-cell imaging showed that the WM treatment significantly decreased GFP signal intensity, even in cells exposed to Abe and Vac (Supplementary Figs. 5A–B). To clarify whether increases in PI(3)P levels induced by the Abe and Vac treatment were involved in accelerating autophagic flux, SH-SY5Y cells stably expressing GFP-LC3-RFP-LC3ΔG were exposed to vehicle, Abe, Vac, WM, Abe plus WM, or Vac plus WM. Abe and Vac significantly decreased the GFP/RFP ratio, as shown in Fig. 1A and B. On the other hand, the GFP/RFP ratios in cells exposed to WM, Abe plus WM, and Vac plus WM were similar to that in vehicle-treated cells. The GRP/RFP ratio was significantly higher in cells exposed to Abe plus WM than in cells exposed to Abe (Supplementary Figs. 6A–B). These results indicate that increases in the formation of PI(3)P induced by Abe and Vac were associated with the acceleration of autophagic flux. To obtain further insights into whether Abe and Vac suppressed the accumulation of aggregate-prone TDP-43 through the formation of PI(3)P, HeLa cells transfected with TDP-43 aa: 162–414 for 24 h were exposed to vehicle, Abe, Vac, WM, Abe plus WM, or Vac plus WM for 6 h. The Abe and Vac treatment for 6 h significantly suppressed the accumulation of TDP-43 aa: 162–414, whereas the WM, Abe plus WM and Vac plus WM treatment did not (Fig. 6E and F), suggesting that WM partially counteracted the suppressive effects of Abe and Vac on aggregate-prone TDP-43 accumulation. Collectively, these results indicate that Abe and Vac inhibited aggregate-prone TDP-43 accumulation by facilitating autophagic flux via a PI(3)P-associated mechanism.

## 4 Discussion

In patients with ALS and FTLD-TDP, disease-causing gene mutations have been shown to suppress autophagy via a number of pathways [2,26]. Autosomal dominant ALS-linked profilin 1 mutants formed aggregates that co-localized with LC3 and decreased the level of the autophagosome marker LC3-II [10]. Progranulin (PGRN), an insufficiency of which due to loss-of-function mutations in the PGRN gene causes FTLD-TDP, has been shown to regulate autophagic flux [13,27,28]. Since a loss of autophagic activity is known to increase the accumulation of aggregate-prone TDP-43 [6], the acceleration of autophagic flux has potential in the treatment of ALS and FTLD-TDP.

The present results suggest that Abe and Vac decreased pathologically aggregated TDP-43 by accelerating omegasome and autolysosome formation in neuroblastoma cells, supporting our hypothesis that Abe and Vac suppress aggregate-prone TDP-43 accumulation via accelerating autophagic flux. The decrease in LAMP1-GFP levels by Abe and Vac is likely due to a fusion of multiple lysosomes onto an autophagosome [29]. On the other hand, we found several differences between the actions of Abe and Vac in degradation of GFP-tagged C-terminal fragment of TDP-43 and regulation of autophagic flux. Abe, but not Vac, significantly suppressed TDP-43 aa: 162–414 accumulation in SH-SY5Y cells, and Abe was also capable of suppressing TDP-43 aa: 162–414 levels in ATG16L1-KO cells. These results suggest that Abe activates other degradation system in addition to autophagy. The previous study showed that CDK4/6 inhibition facilitates ubiquitination of ZEB1, a key molecule in tumor metastasis, via suppressing activation of the deubiquitinase USP51 [30]. Thus, Abe possibly facilitates degradation of GFP-tagged C-terminal fragment of TDP-43 via the ubiquitin-proteasome pathway [31]. Similarly, Abe and Vac facilitated autophagic flux and increased autolysosome formation, but there were differences in the omegasome formation and autophagosome numbers. Abe significantly increased the omegasome marker accumulation, whereas Vac showed a subtle effect on the omegasome formation. CDK4/6 inhibition is known to facilitate the autophagy-lysosome pathway by activating transcription factors TFEB and TFE3 [32], and suppression of mTORC1 activity via AMPK [33] and TSC2 [34] activation. On the other hand, a PIKfyve inhibitor APY0201 was identified as a molecule that up-regulates the autophagy-lysosome pathway via TFEB activation [35]. Although it remains to be fully determined whether Abe or Vac influences TDP-43 synthesis, Abe likely have broader targets compared to Vac, and the off-target effects provide a synergy on facilitating TDP-43 degradation via the autophagy-lysosome pathway and the ubiquitin-proteasome pathway. Further investigation is warranted for determine the underlying mechanisms.

Abe and Vac facilitated autophagic flux by increasing PI(3)P levels. PI(3)P is an autophagy inducer [16]. On the other hand, the hyperactivation of VPS34 was previously shown to induce the extensive formation of vacuole-like autolysosomes [22]. The results of our reporter assay using the fluorescent protein-tagged 2 × FYVE domain indicated that PI(3)P localizes to vacuole-like autolysosomes. The increased PI(3)P level in lysosomes possibly facilitate the autophagic flux upon Abe/Vac treatment due to the activated autophagosome formation and autophagosome-lysosome fusion.

PIKfyve synthesizes PI(3,5)P2 from PI(3)P [16]. Abe may inhibit PIKfyve as well as the PIKfyve inhibitor Vac. PIKfyve mainly localizes to endosomes or lysosomes, at which it regulates recycling of membrane proteins back into the plasma membrane through a retriever complex comprising VPS29, VPS35L, and VPS26C [20]. However, it remains unknown whether this retriever complex regulates autophagosome-lysosome fusion. Further studies are needed to elucidate the mechanisms underlying Abe-/Vac-induced autolysosome formation. On the other hand, a previous study indicated that a PIKfyve inhibitor time-dependently suppressed autophagic flux [36]. Vacuoles induced by PIKfyve inhibitors were formed by autophagosomes and lysosomes [7,22], and the inhibition of PIKfyve decreased lysosome numbers [37]. Therefore, the depletion of autophagosomes or lysosomes due to excessive autophagosome-lysosome fusion through the inhibition of PIKfyve may suppress autophagic flux.

Although TDP-43 is a nuclear protein, aggregate-prone TDP-43 is mainly degraded by autophagy [5,6]. Since the suppression of autophagic flux increases aggregate-prone TDP-43 accumulation [7], the Abe and Vac treatment may have promoted the degradation of aggregate-prone TDP-43 that accumulated in patients with suppressed autophagic flux. Abe has been approved for the treatment of breast cancer and penetrates the blood-brain barrier [38]. Although future studies using animal models are required to clarify whether Abe suppresses TDP-43 accumulation by promoting autophagic flux, the present results indicate that Abe is a suitable candidate for the treatment of ALS and FTLD-TDP through its acceleration of autophagic flux.

The present results indicate that the Abe and Vac treatment decreased aggregate-prone TDP-43 accumulation by facilitating autophagic flux. Although further studies are required to clarify the mechanisms responsible, we propose a novel mechanism in which the regulation of autophagic flux is associated with the formation of PI(3)P. Drug therapy using a suitable concentration of Abe may reduce aggregate-prone proteins and inhibit the progression of TDP-43-associated neurodegenerative diseases.

## CRediT authorship contribution statement

**Yoshinori Tanaka:** Project administration, Methodology, Investigation, Funding acquisition, Data curation, Conceptualization, Resources, Supervision, Validation, Visualization, Writing – original draft, Writing – review & editing. **Lina Kozuma:** Investigation. **Hirotsugu Hino:** Writing – review & editing, Resources, Validation. **Kosuke Takeya:** Writing – review & editing. **Masumi Eto:** Writing – review & editing.

## Declaration of competing interest

The authors declare that they have no known competing financial interests or personal relationships that could have appeared to influence the work reported in this paper.
