# Supplementary material for: Abemaciclib and Vacuolin-1 decrease aggregate-prone TDP-43 accumulation by accelerating autophagic flux
Source: Biochem Biophys Rep. 2024 Apr 1;38:101705. doi: 10.1016/j.bbrep.2024.101705 (PMC11001778; doi:10.1016/j.bbrep.2024.101705)
Supplement: Multimedia component 1 [file mmc1.pptx]

## Slide 1
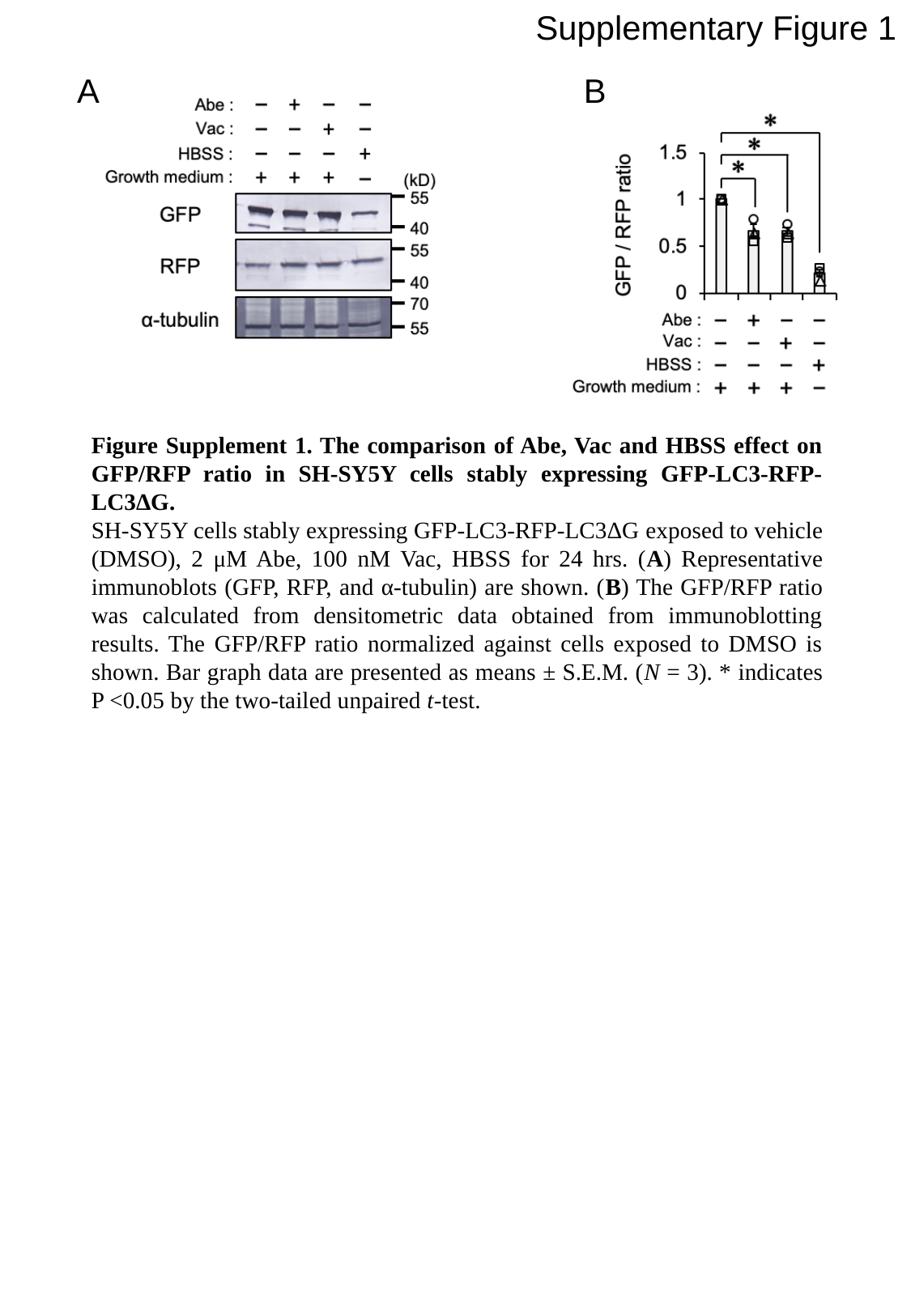

Supplementary Figure 1
A
B
Figure Supplement 1. The comparison of Abe, Vac and HBSS effect on GFP/RFP ratio in SH-SY5Y cells stably expressing GFP-LC3-RFP-LC3ΔG.
SH-SY5Y cells stably expressing GFP-LC3-RFP-LC3ΔG exposed to vehicle (DMSO), 2 μM Abe, 100 nM Vac, HBSS for 24 hrs. (A) Representative immunoblots (GFP, RFP, and α-tubulin) are shown. (B) The GFP/RFP ratio was calculated from densitometric data obtained from immunoblotting results. The GFP/RFP ratio normalized against cells exposed to DMSO is shown. Bar graph data are presented as means ± S.E.M. (N = 3). * indicates P <0.05 by the two-tailed unpaired t-test.
