# Supplementary material for: Abemaciclib and Vacuolin-1 decrease aggregate-prone TDP-43 accumulation by accelerating autophagic flux
Source: Biochem Biophys Rep. 2024 Apr 1;38:101705. doi: 10.1016/j.bbrep.2024.101705 (PMC11001778; doi:10.1016/j.bbrep.2024.101705)
Supplement: Multimedia component 2 [file mmc2.pptx]

## Slide 1
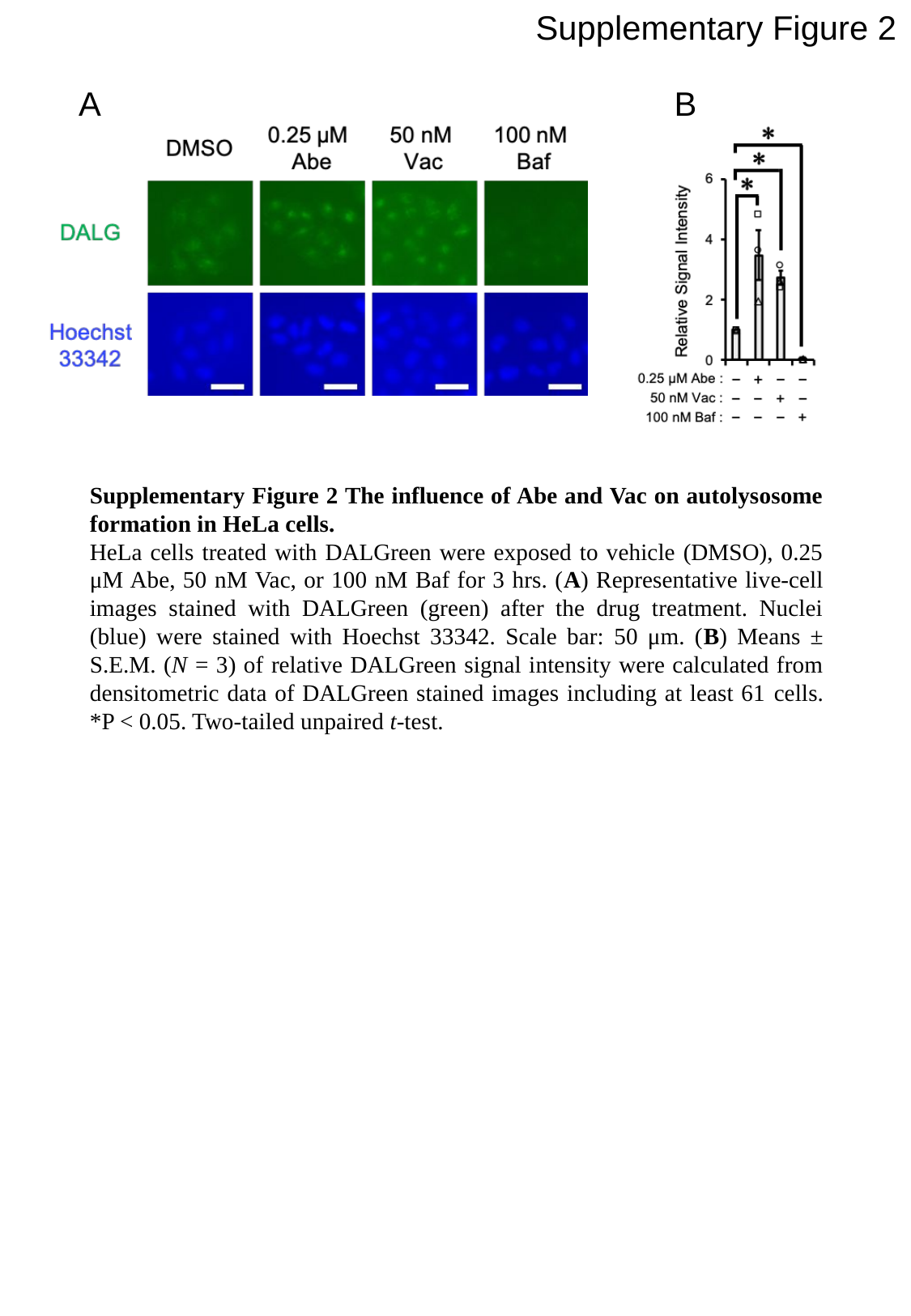

Supplementary Figure 2
A
B
Supplementary Figure 2 The influence of Abe and Vac on autolysosome formation in HeLa cells.
HeLa cells treated with DALGreen were exposed to vehicle (DMSO), 0.25 μM Abe, 50 nM Vac, or 100 nM Baf for 3 hrs. (A) Representative live-cell images stained with DALGreen (green) after the drug treatment. Nuclei (blue) were stained with Hoechst 33342. Scale bar: 50 μm. (B) Means ± S.E.M. (N = 3) of relative DALGreen signal intensity were calculated from densitometric data of DALGreen stained images including at least 61 cells. *P < 0.05. Two-tailed unpaired t-test.
