# Supplementary material for: Abemaciclib and Vacuolin-1 decrease aggregate-prone TDP-43 accumulation by accelerating autophagic flux
Source: Biochem Biophys Rep. 2024 Apr 1;38:101705. doi: 10.1016/j.bbrep.2024.101705 (PMC11001778; doi:10.1016/j.bbrep.2024.101705)
Supplement: Multimedia component 3 [file mmc3.pptx]

## Slide 1
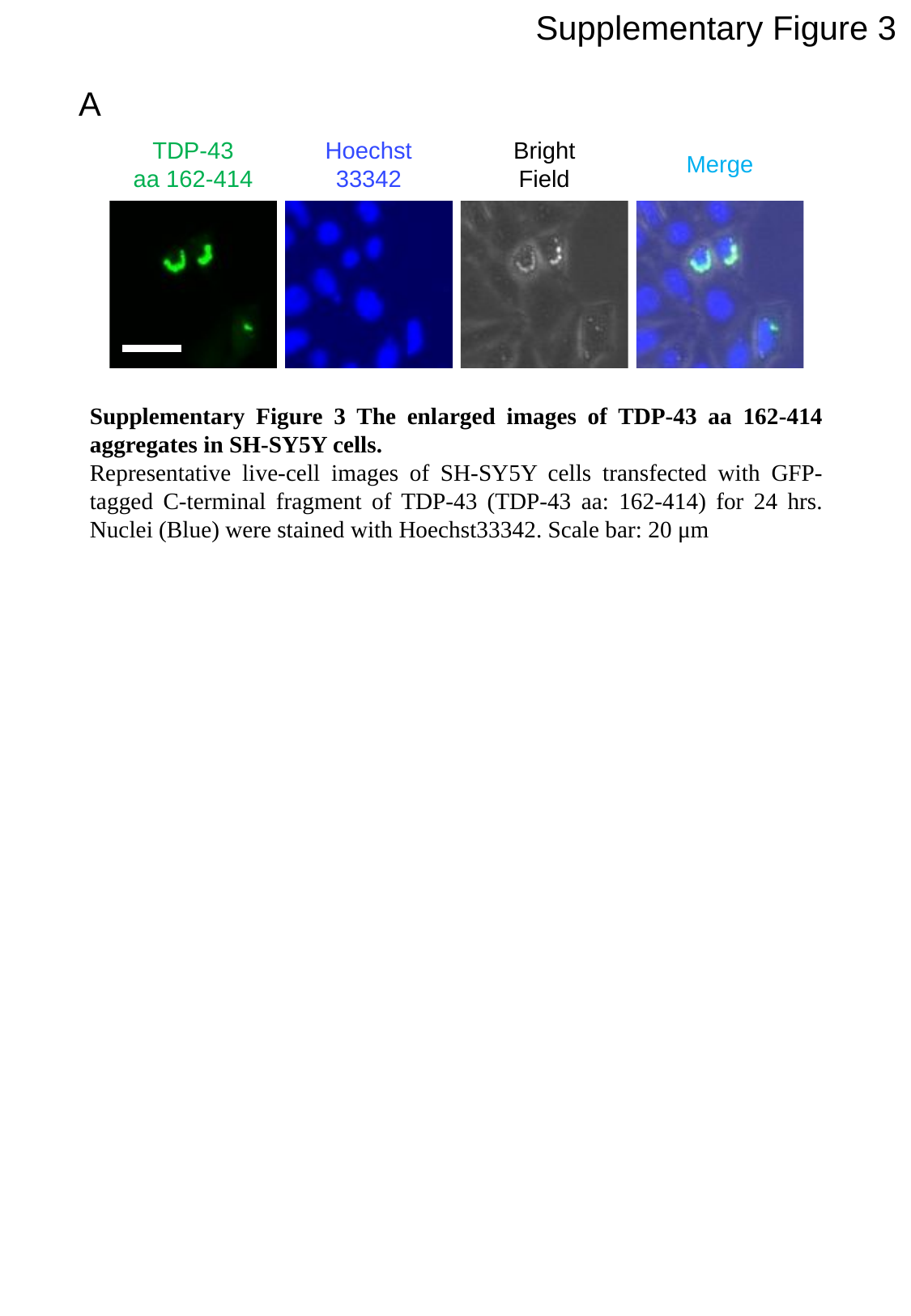

Supplementary Figure 3
A
TDP-43
aa 162-414
Hoechst
33342
Bright
Field
Merge
Supplementary Figure 3 The enlarged images of TDP-43 aa 162-414 aggregates in SH-SY5Y cells.
Representative live-cell images of SH-SY5Y cells transfected with GFP-tagged C-terminal fragment of TDP-43 (TDP-43 aa: 162-414) for 24 hrs. Nuclei (Blue) were stained with Hoechst33342. Scale bar: 20 μm
