# Supplementary material for: Abemaciclib and Vacuolin-1 decrease aggregate-prone TDP-43 accumulation by accelerating autophagic flux
Source: Biochem Biophys Rep. 2024 Apr 1;38:101705. doi: 10.1016/j.bbrep.2024.101705 (PMC11001778; doi:10.1016/j.bbrep.2024.101705)
Supplement: Multimedia component 4 [file mmc4.pptx]

## Slide 1
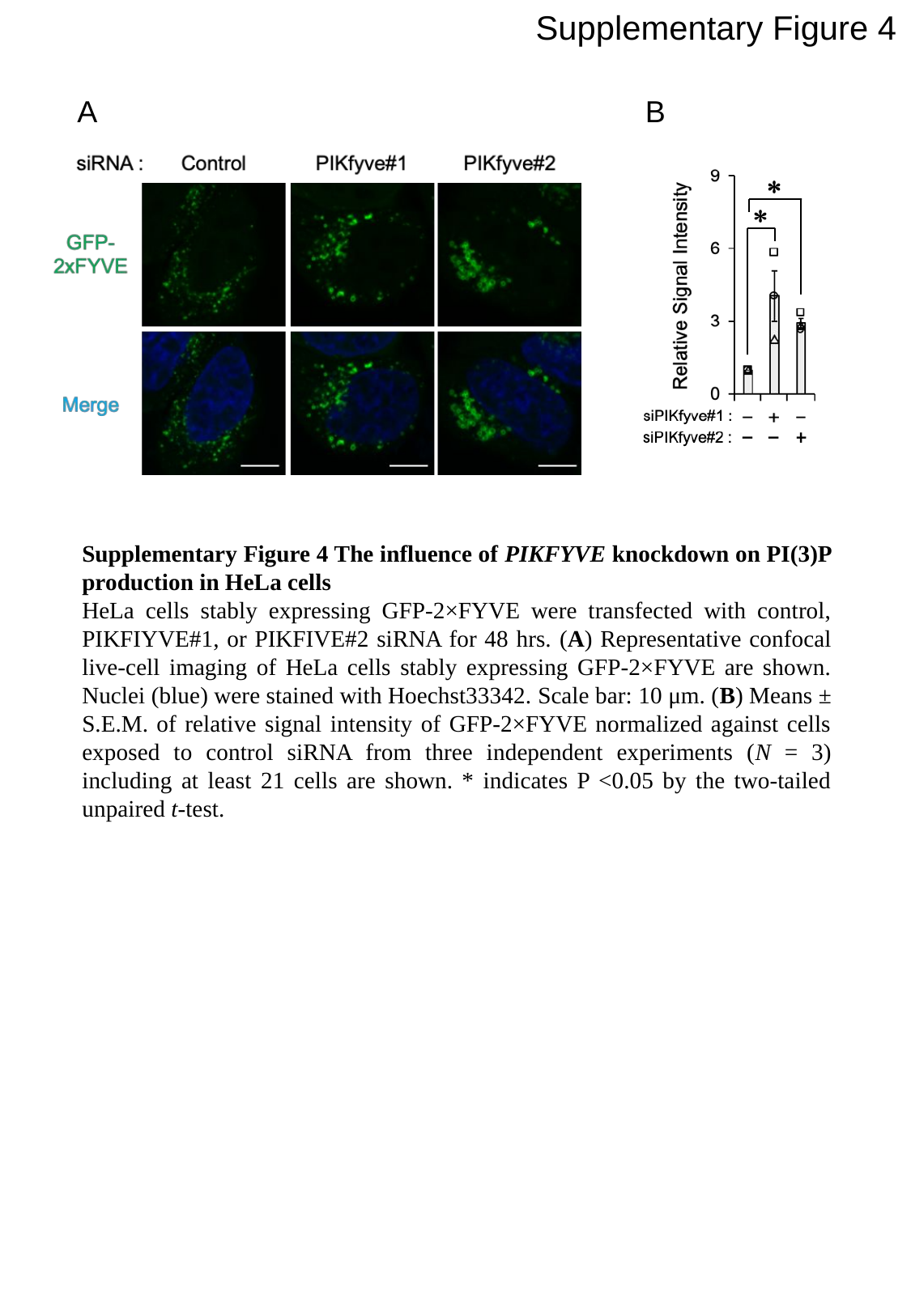

Supplementary Figure 4
A
B
Supplementary Figure 4 The influence of PIKFYVE knockdown on PI(3)P production in HeLa cells
HeLa cells stably expressing GFP-2×FYVE were transfected with control, PIKFIYVE#1, or PIKFIVE#2 siRNA for 48 hrs. (A) Representative confocal live-cell imaging of HeLa cells stably expressing GFP-2×FYVE are shown. Nuclei (blue) were stained with Hoechst33342. Scale bar: 10 μm. (B) Means ± S.E.M. of relative signal intensity of GFP-2×FYVE normalized against cells exposed to control siRNA from three independent experiments (N = 3) including at least 21 cells are shown. * indicates P <0.05 by the two-tailed unpaired t-test.
