# Supplementary material for: Abemaciclib and Vacuolin-1 decrease aggregate-prone TDP-43 accumulation by accelerating autophagic flux
Source: Biochem Biophys Rep. 2024 Apr 1;38:101705. doi: 10.1016/j.bbrep.2024.101705 (PMC11001778; doi:10.1016/j.bbrep.2024.101705)
Supplement: Multimedia component 5 [file mmc5.pptx]

## Slide 1
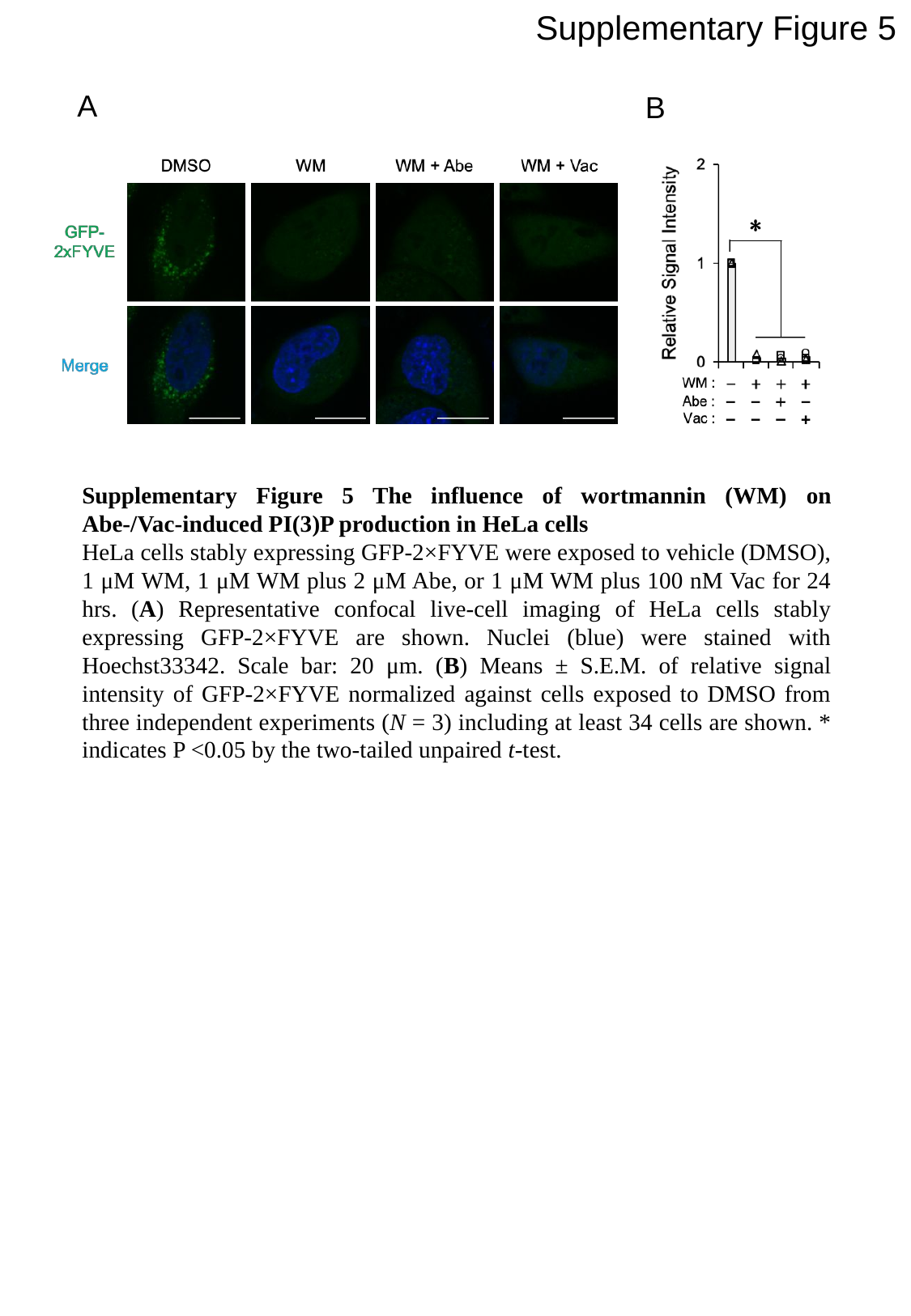

Supplementary Figure 5
A
B
Supplementary Figure 5 The influence of wortmannin (WM) on Abe-/Vac-induced PI(3)P production in HeLa cells
HeLa cells stably expressing GFP-2×FYVE were exposed to vehicle (DMSO), 1 μM WM, 1 μM WM plus 2 μM Abe, or 1 μM WM plus 100 nM Vac for 24 hrs. (A) Representative confocal live-cell imaging of HeLa cells stably expressing GFP-2×FYVE are shown. Nuclei (blue) were stained with Hoechst33342. Scale bar: 20 μm. (B) Means ± S.E.M. of relative signal intensity of GFP-2×FYVE normalized against cells exposed to DMSO from three independent experiments (N = 3) including at least 34 cells are shown. * indicates P <0.05 by the two-tailed unpaired t-test.
