# Supplementary material for: Abemaciclib and Vacuolin-1 decrease aggregate-prone TDP-43 accumulation by accelerating autophagic flux
Source: Biochem Biophys Rep. 2024 Apr 1;38:101705. doi: 10.1016/j.bbrep.2024.101705 (PMC11001778; doi:10.1016/j.bbrep.2024.101705)
Supplement: Multimedia component 6 [file mmc6.pptx]

## Slide 1
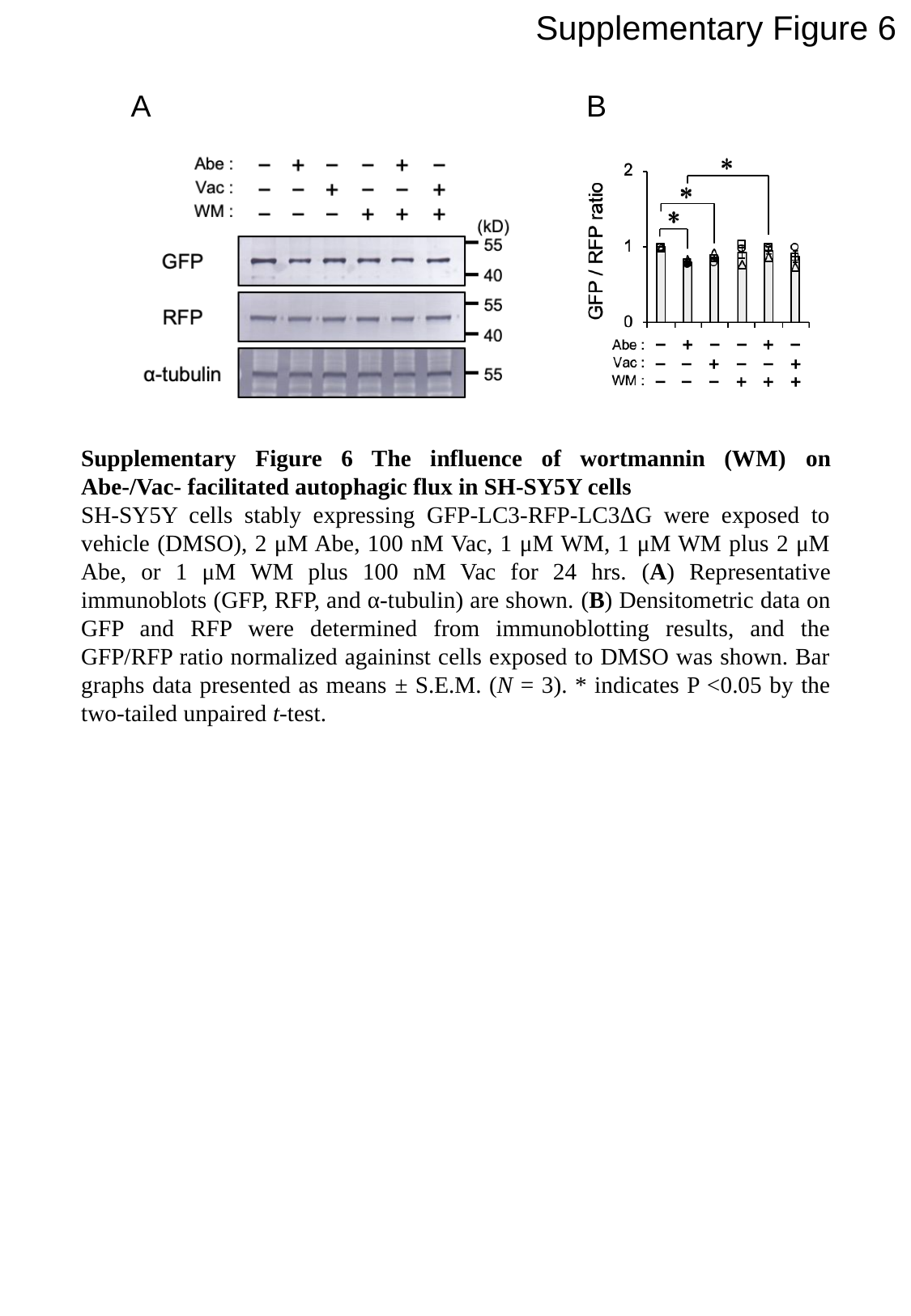

Supplementary Figure 6
A
B
Supplementary Figure 6 The influence of wortmannin (WM) on Abe-/Vac- facilitated autophagic flux in SH-SY5Y cells
SH-SY5Y cells stably expressing GFP-LC3-RFP-LC3ΔG were exposed to vehicle (DMSO), 2 μM Abe, 100 nM Vac, 1 μM WM, 1 μM WM plus 2 μM Abe, or 1 μM WM plus 100 nM Vac for 24 hrs. (A) Representative immunoblots (GFP, RFP, and α-tubulin) are shown. (B) Densitometric data on GFP and RFP were determined from immunoblotting results, and the GFP/RFP ratio normalized againinst cells exposed to DMSO was shown. Bar graphs data presented as means ± S.E.M. (N = 3). * indicates P <0.05 by the two-tailed unpaired t-test.
