# Supplementary material for: Abemaciclib and Vacuolin-1 decrease aggregate-prone TDP-43 accumulation by accelerating autophagic flux
Source: Biochem Biophys Rep. 2024 Apr 1;38:101705. doi: 10.1016/j.bbrep.2024.101705 (PMC11001778; doi:10.1016/j.bbrep.2024.101705)
Supplement: Multimedia component 8 [file mmc8.pptx]

## Slide 1
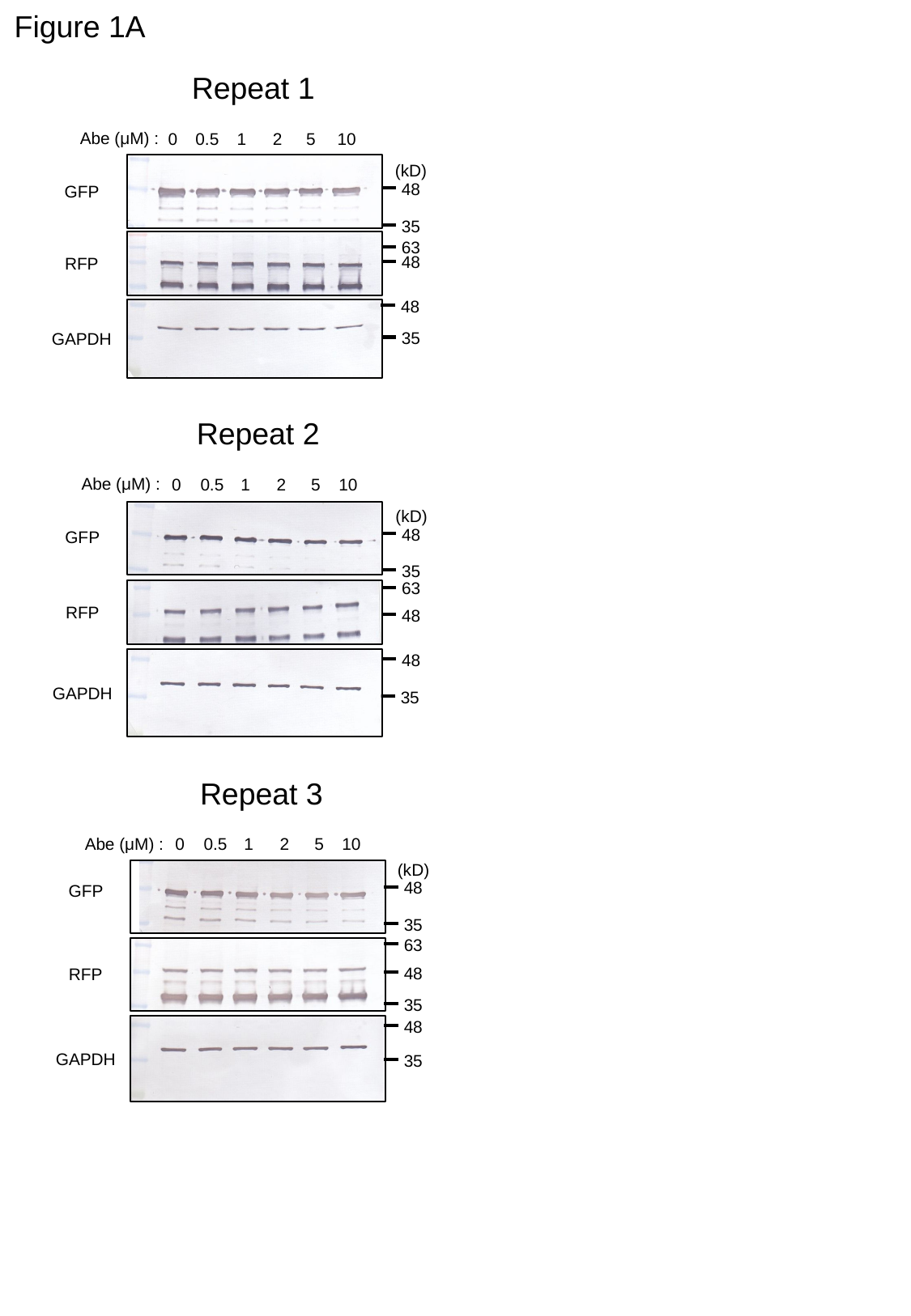

Figure 1A
Repeat 1
Abe (μM) :
0
0.5
1
2
5
10
(kD)
48
GFP
35
63
48
RFP
48
35
GAPDH
Repeat 2
Abe (μM) :
0
0.5
1
2
5
10
(kD)
48
GFP
35
63
RFP
48
48
GAPDH
35
Repeat 3
Abe (μM) :
0
0.5
1
2
5
10
(kD)
48
GFP
35
63
48
RFP
35
48
GAPDH
35

## Slide 2
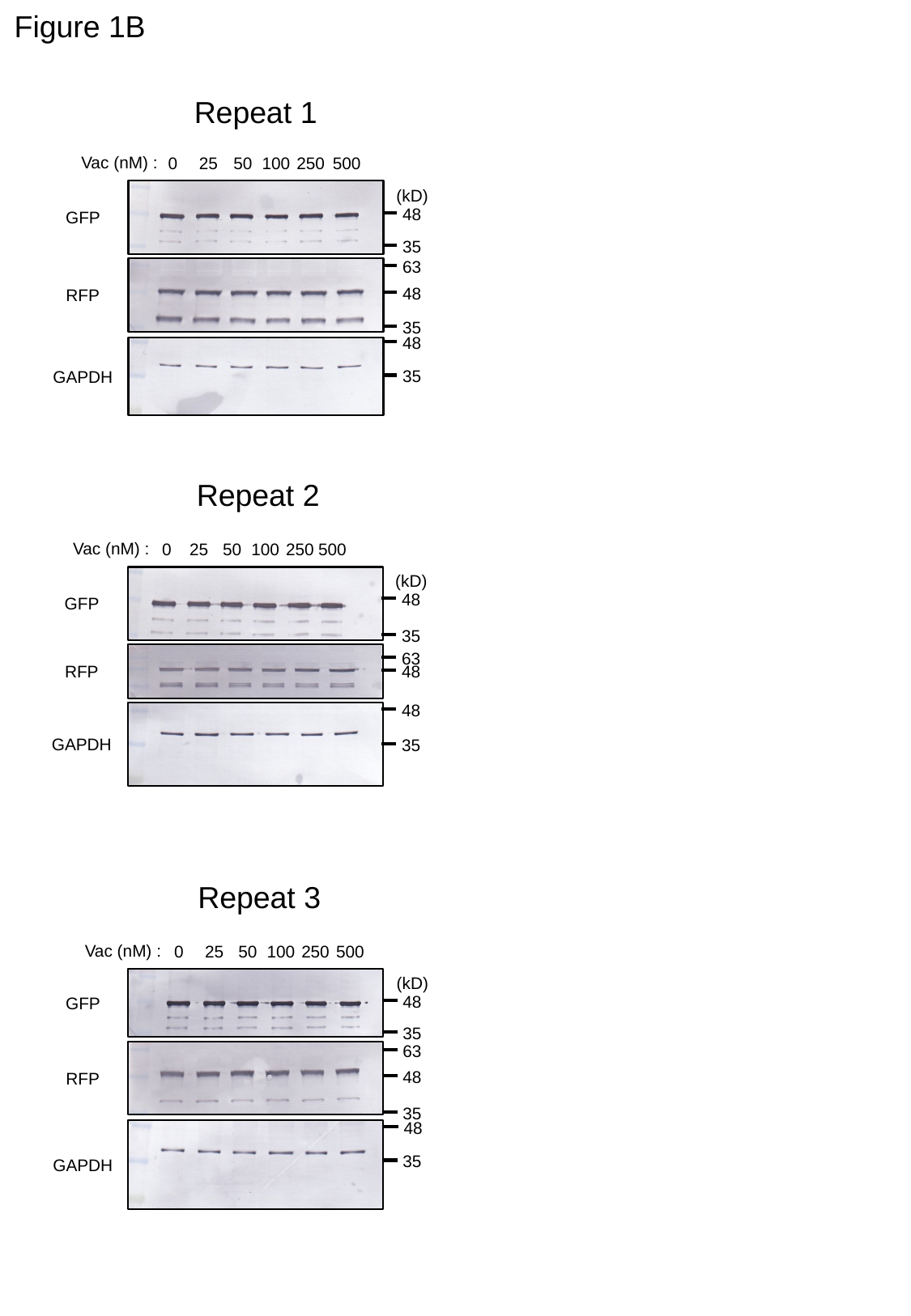

Figure 1B
Repeat 1
Vac (nM) :
0
25
50
100
250
500
(kD)
48
GFP
35
63
48
RFP
35
48
35
GAPDH
Repeat 2
Vac (nM) :
0
25
50
100
250
500
(kD)
48
GFP
35
63
48
RFP
48
GAPDH
35
Repeat 3
Vac (nM) :
0
25
50
100
250
500
(kD)
48
GFP
35
63
48
RFP
35
48
35
GAPDH

## Slide 3
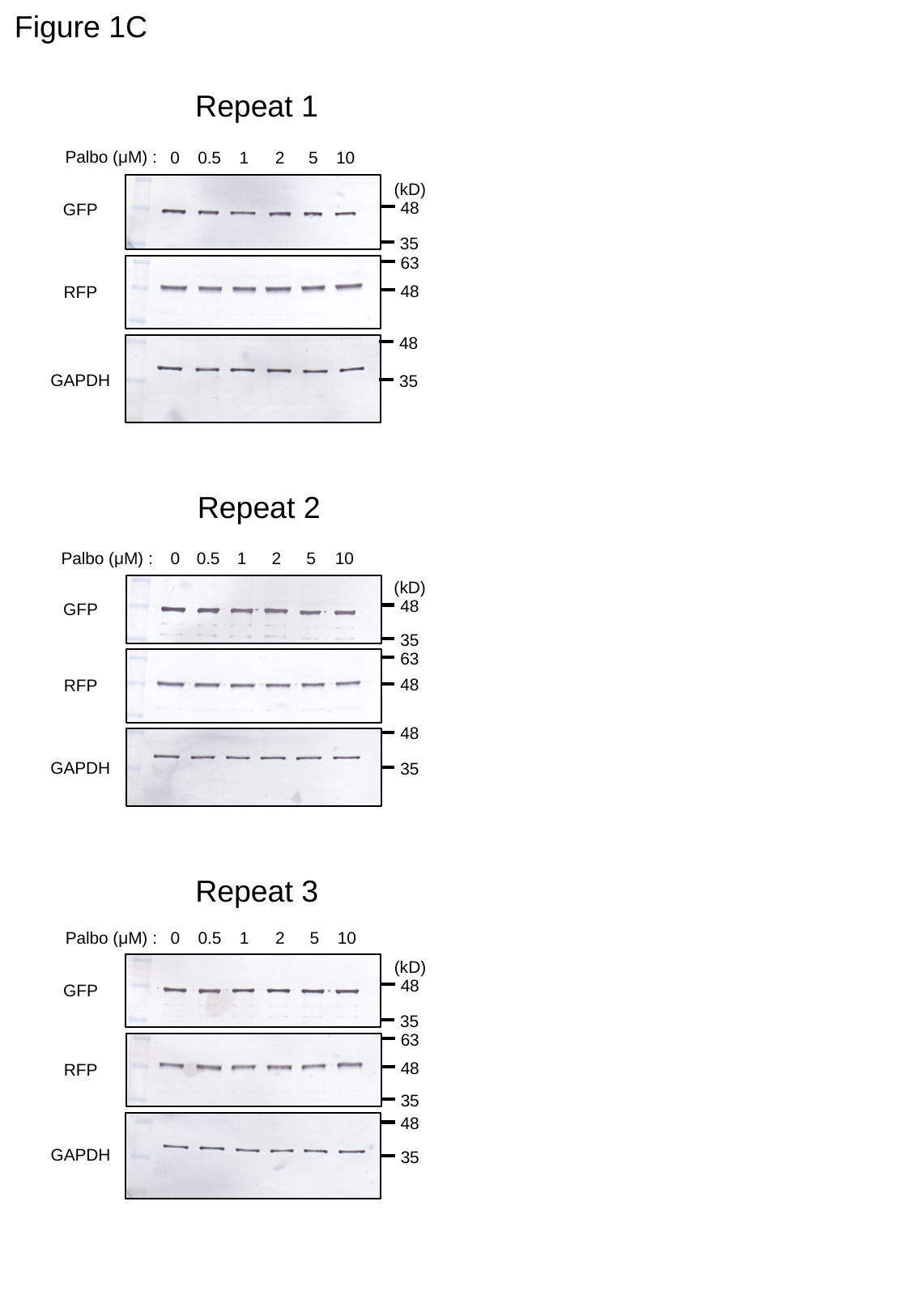

Figure 1C
Repeat 1
Palbo (μM) :
0
0.5
1
2
5
10
(kD)
48
GFP
35
63
48
RFP
48
GAPDH
35
Repeat 2
Palbo (μM) :
0
0.5
1
2
5
10
(kD)
48
GFP
35
63
48
RFP
48
GAPDH
35
Repeat 3
Palbo (μM) :
0
0.5
1
2
5
10
(kD)
48
GFP
35
63
48
RFP
35
48
GAPDH
35

## Slide 4
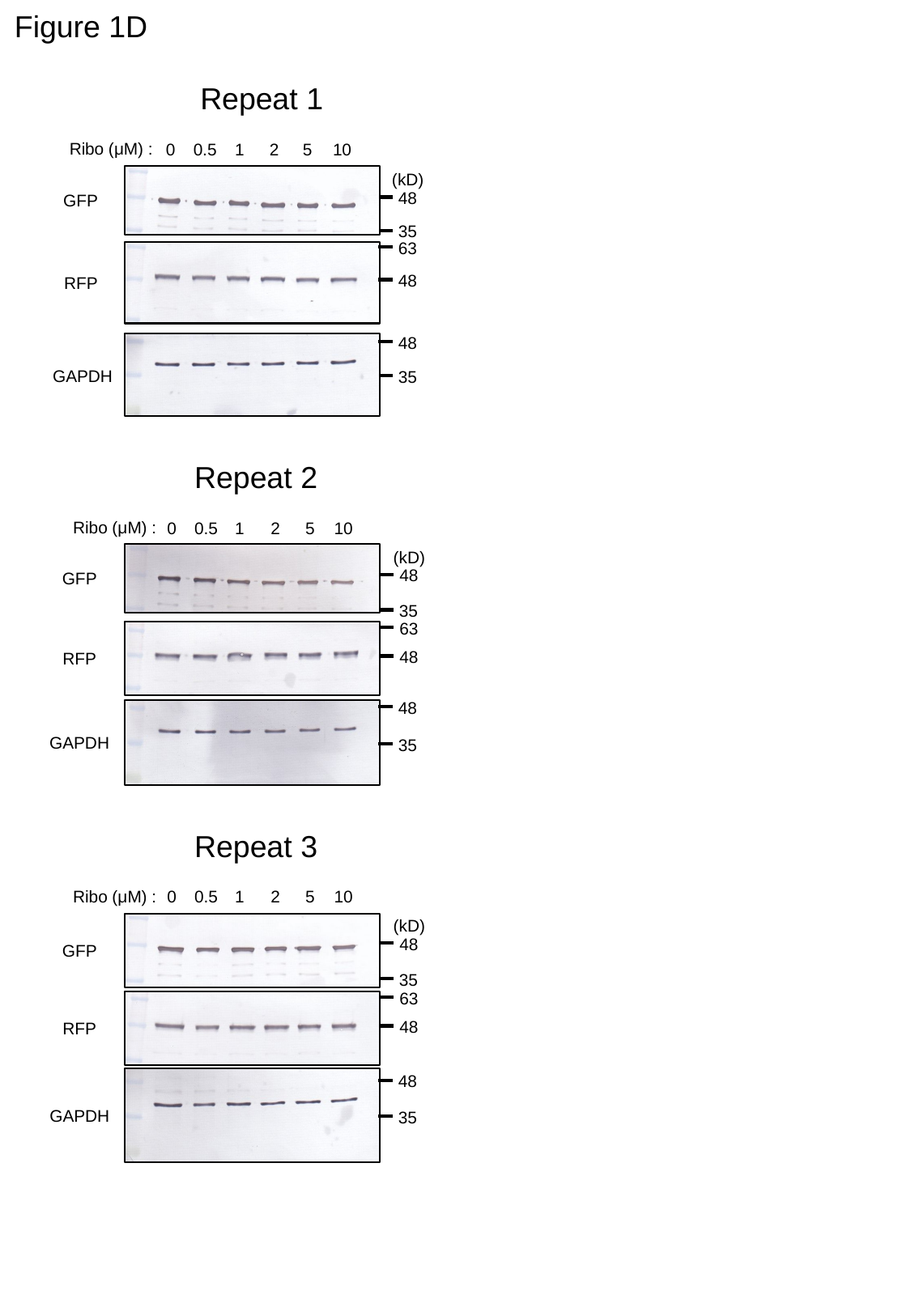

Figure 1D
Repeat 1
Ribo (μM) :
0
0.5
1
2
5
10
(kD)
48
GFP
35
63
48
RFP
48
GAPDH
35
Repeat 2
Ribo (μM) :
0
0.5
1
2
5
10
(kD)
48
GFP
35
63
48
RFP
48
GAPDH
35
Repeat 3
Ribo (μM) :
0
0.5
1
2
5
10
(kD)
48
GFP
35
63
48
RFP
48
GAPDH
35

## Slide 5
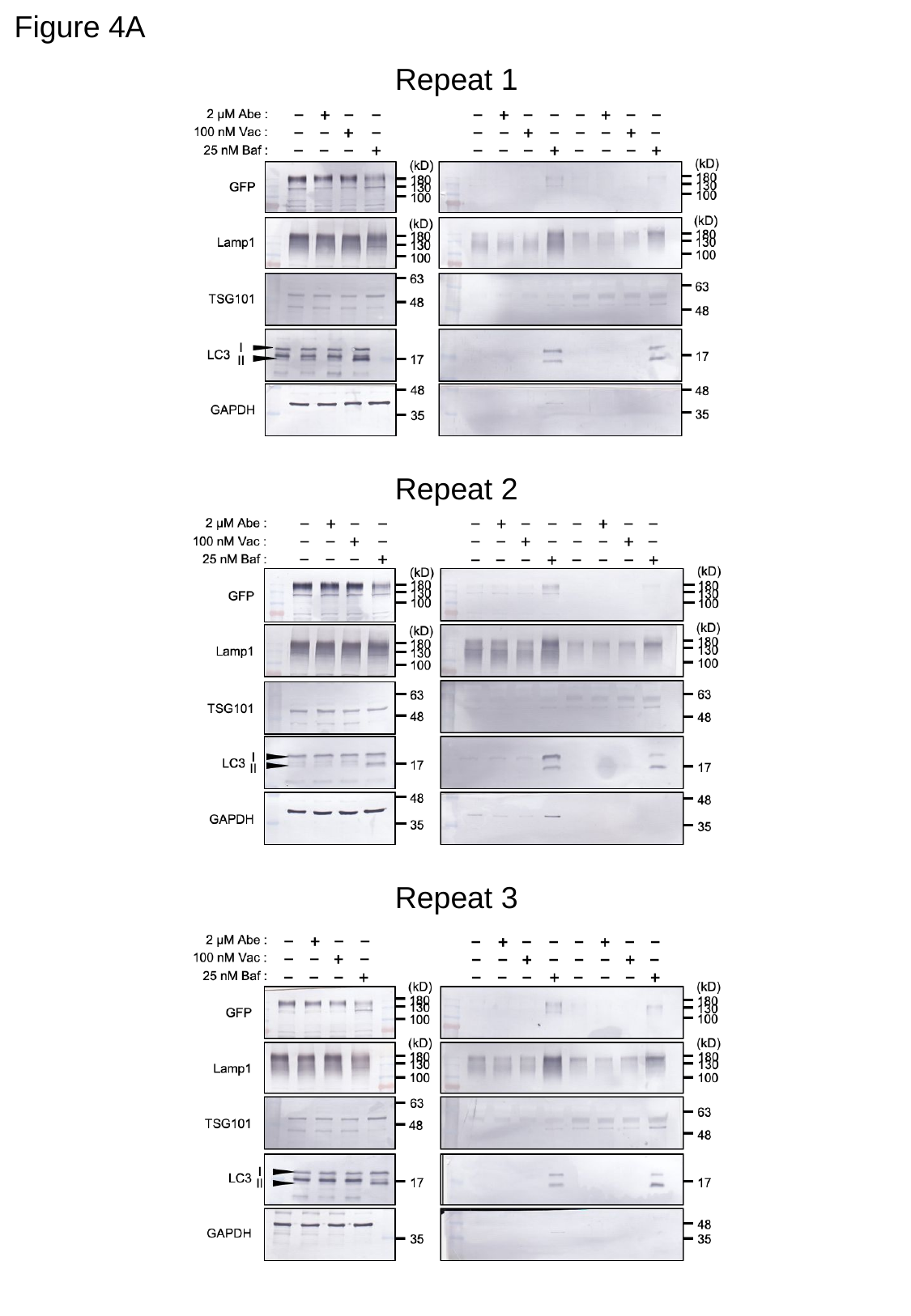

Figure 4A
Repeat 1
Repeat 2
Repeat 3

## Slide 6
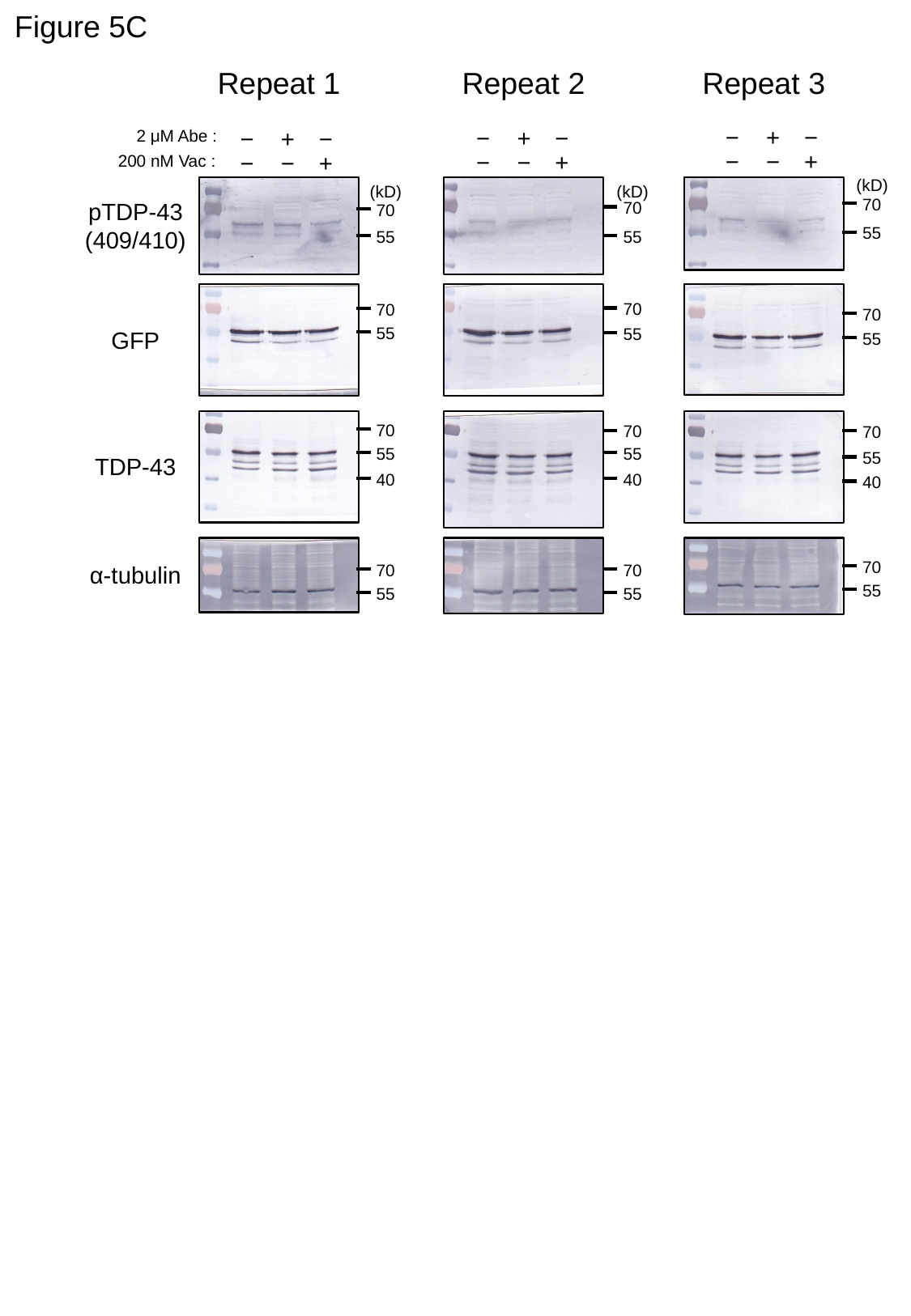

Figure 5C
Repeat 1
Repeat 2
Repeat 3
−
+
−
−
−
+
−
+
−
−
−
+
−
+
−
2 μM Abe :
−
−
+
200 nM Vac :
(kD)
(kD)
(kD)
70
70
pTDP-43
(409/410)
70
55
55
55
70
70
70
55
55
GFP
55
70
70
70
55
55
55
TDP-43
40
40
40
70
70
70
α-tubulin
55
55
55

## Slide 7
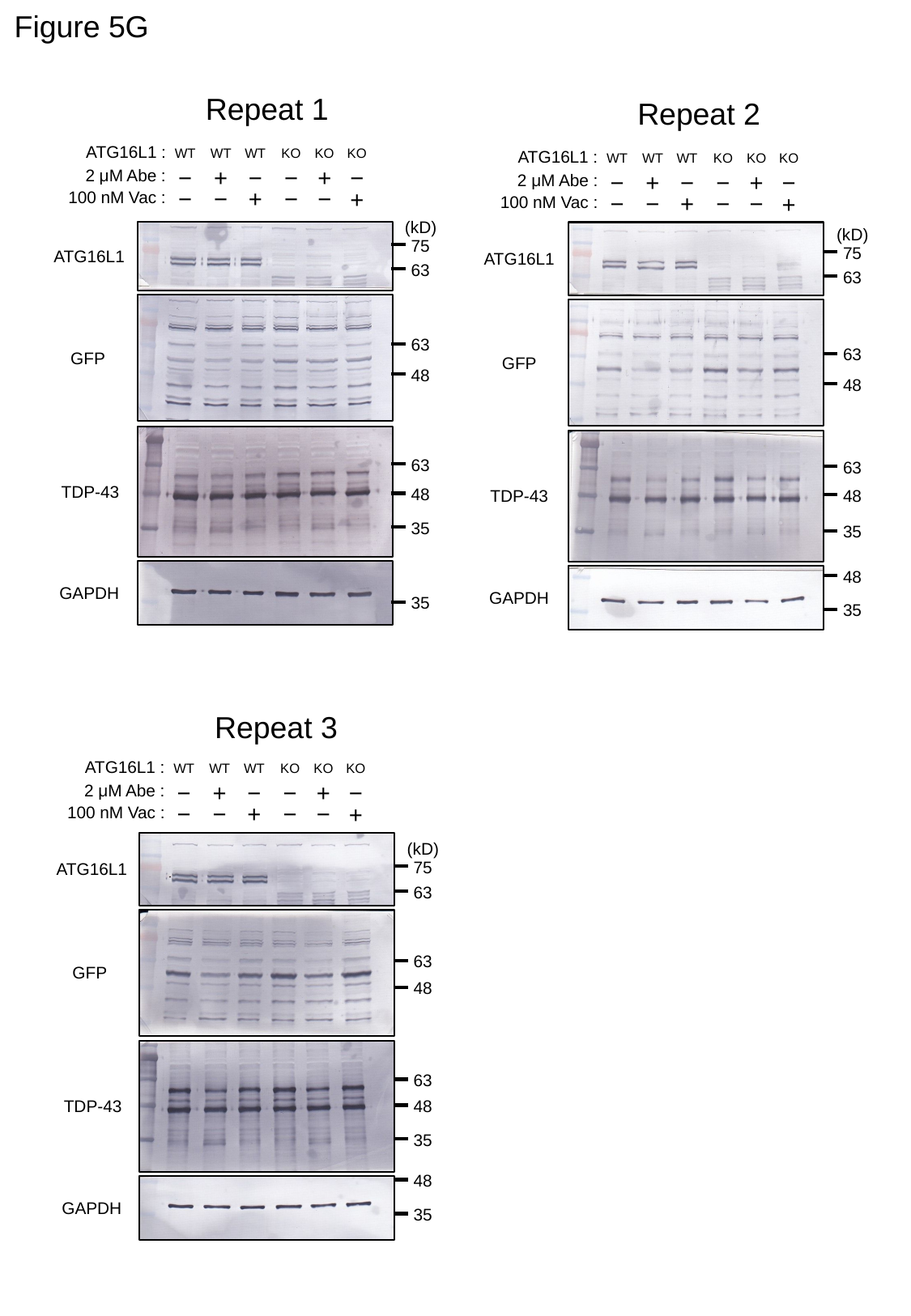

Figure 5G
Repeat 1
Repeat 2
ATG16L1 :
WT
WT
WT
KO
KO
KO
−
−
−
−
+
+
2 μM Abe :
−
−
−
−
+
+
100 nM Vac :
ATG16L1 :
WT
WT
WT
KO
KO
KO
−
−
−
−
+
+
2 μM Abe :
−
−
−
−
+
+
100 nM Vac :
(kD)
75
(kD)
75
ATG16L1
ATG16L1
63
63
63
63
GFP
GFP
48
48
63
63
TDP-43
48
48
TDP-43
35
35
48
35
GAPDH
GAPDH
35
Repeat 3
ATG16L1 :
WT
WT
WT
KO
KO
KO
−
−
−
−
+
+
2 μM Abe :
−
−
−
−
+
+
100 nM Vac :
(kD)
75
ATG16L1
63
63
GFP
48
63
48
TDP-43
35
48
GAPDH
35

## Slide 8
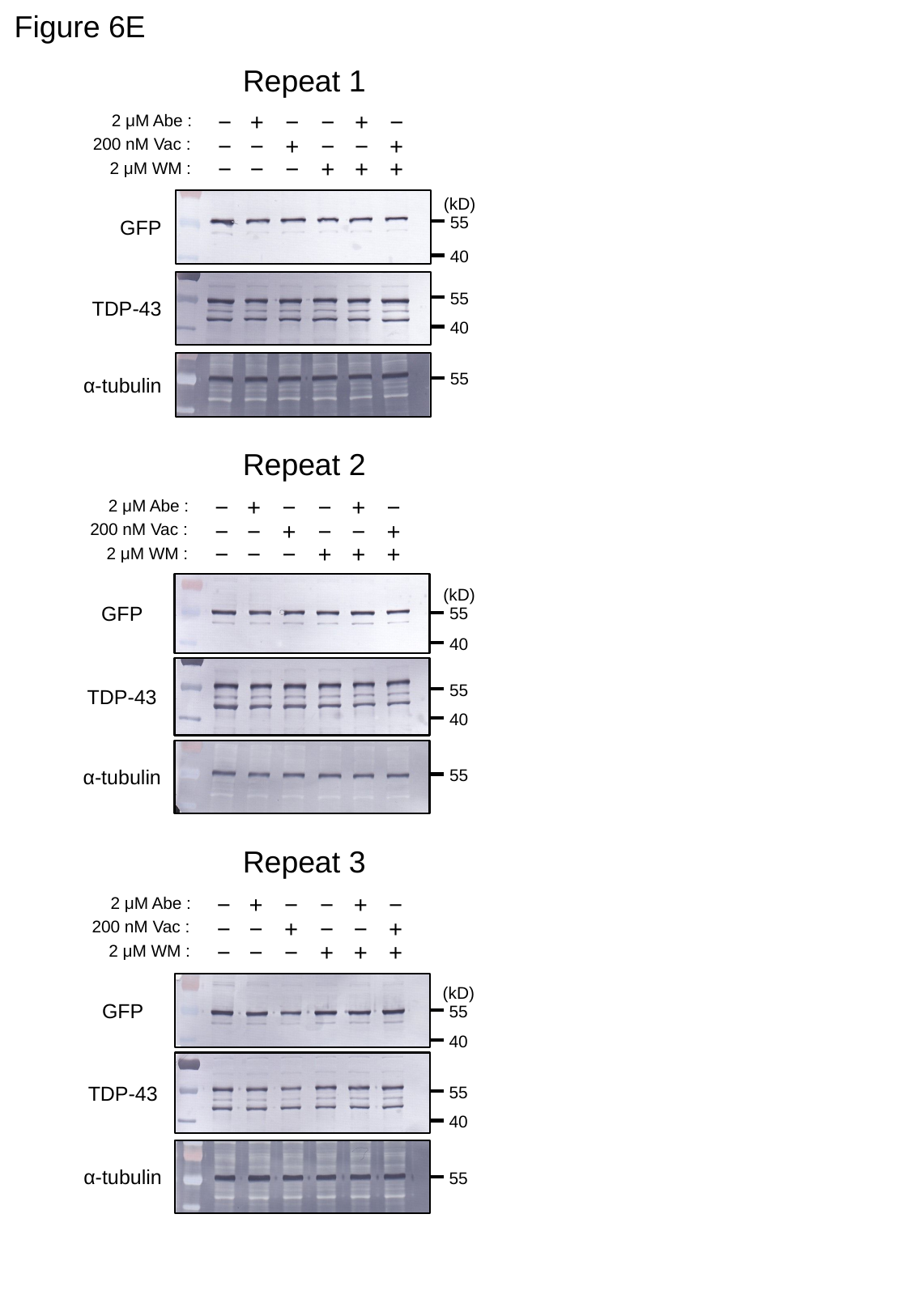

Figure 6E
Repeat 1
−
+
−
−
+
−
2 μM Abe :
−
−
+
−
−
+
200 nM Vac :
−
−
−
+
+
+
2 μM WM :
(kD)
55
GFP
40
55
TDP-43
40
55
α-tubulin
Repeat 2
−
+
−
−
+
−
2 μM Abe :
−
−
+
−
−
+
200 nM Vac :
−
−
−
+
+
+
2 μM WM :
(kD)
55
GFP
40
55
TDP-43
40
α-tubulin
55
Repeat 3
−
+
−
−
+
−
2 μM Abe :
−
−
+
−
−
+
200 nM Vac :
−
−
−
+
+
+
2 μM WM :
(kD)
55
GFP
40
TDP-43
55
40
α-tubulin
55

## Slide 9
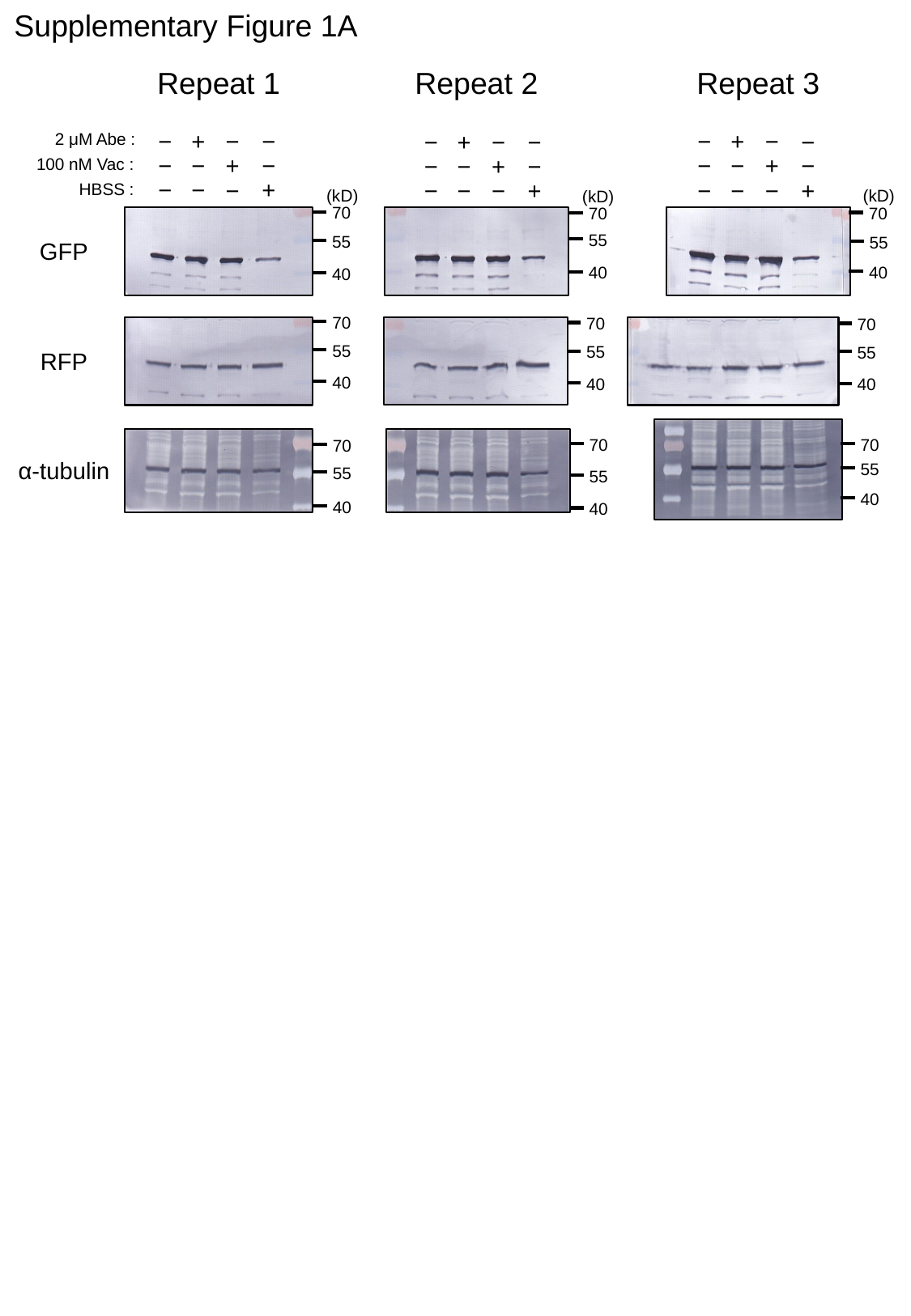

Supplementary Figure 1A
Repeat 1
Repeat 2
Repeat 3
−
+
−
−
2 μM Abe :
−
−
+
−
100 nM Vac :
−
+
−
−
HBSS :
−
+
−
−
−
−
+
−
−
+
−
−
−
+
−
−
−
−
+
−
−
+
−
−
(kD)
(kD)
(kD)
70
70
70
55
55
55
GFP
40
40
40
70
70
70
55
40
55
55
RFP
40
40
70
55
40
70
55
40
70
55
40
α-tubulin

## Slide 10
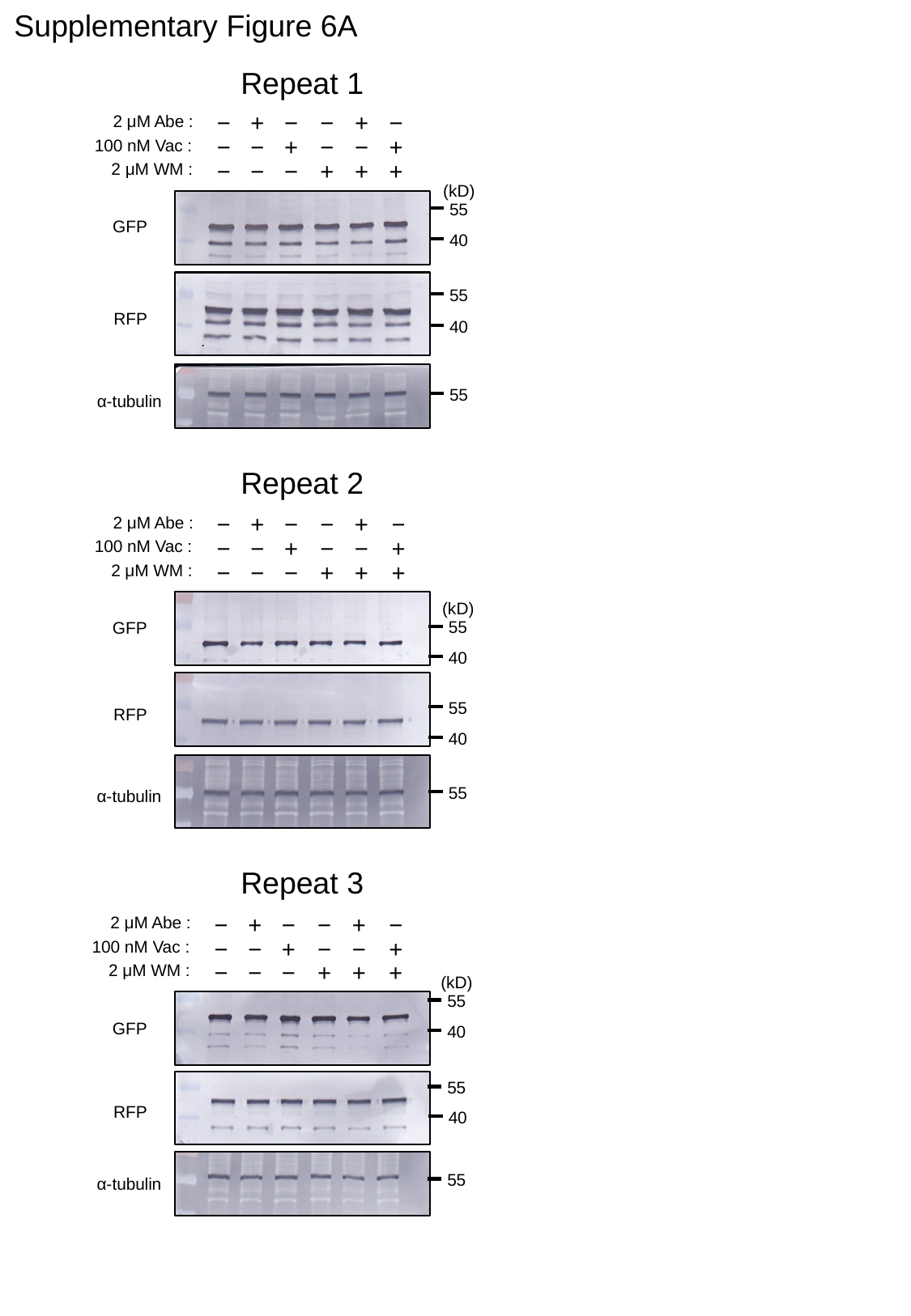

Supplementary Figure 6A
Repeat 1
−
−
+
−
−
+
2 μM Abe :
−
−
+
−
−
+
100 nM Vac :
−
−
−
+
+
+
2 μM WM :
(kD)
55
GFP
40
55
RFP
40
55
α-tubulin
Repeat 2
−
−
+
−
−
+
2 μM Abe :
−
−
+
−
−
+
100 nM Vac :
−
−
−
+
+
+
2 μM WM :
(kD)
55
GFP
40
55
40
RFP
55
α-tubulin
Repeat 3
−
−
+
−
−
+
2 μM Abe :
−
−
+
−
−
+
100 nM Vac :
−
−
−
+
+
+
2 μM WM :
(kD)
55
GFP
40
55
RFP
40
55
α-tubulin
